# Pit and tracheid anatomy explain hydraulic safety but not hydraulic efficiency of 28 conifer species

**DOI:** 10.1093/jxb/erab449

**Published:** 2021-10-09

**Authors:** Yanjun Song, Lourens Poorter, Angelina Horsting, Sylvain Delzon, Frank Sterck

**Affiliations:** 1 Forest Ecology and Forest Management Group, Wageningen University and Research, PO Box 47, 6700 AA, Wageningen, The Netherlands; 2 University of Bordeaux, INRA, UMR BIOGECO, 33450 Talence, France; 3 McGill University, Canada

**Keywords:** Cavitation resistance, conifer species, embolism, hydraulic efficiency, phylogeny, pit sealing, pit size

## Abstract

Conifers face increased drought mortality risks because of drought-induced embolism in their vascular system. Variation in embolism resistance may result from species differences in pit structure and function, as pits control the air seeding between water-transporting conduits. This study quantifies variation in embolism resistance and hydraulic conductivity for 28 conifer species grown in a 50-year-old common garden experiment and assesses the underlying mechanisms. Conifer species with a small pit aperture, high pit aperture resistance, and large valve effect were more resistant to embolism, as they all may reduce air seeding. Surprisingly, hydraulic conductivity was only negatively correlated with tracheid cell wall thickness. Embolism resistance and its underlying pit traits related to pit size and sealing were more strongly phylogenetically controlled than hydraulic conductivity and anatomical tracheid traits. Conifers differed in hydraulic safety and hydraulic efficiency, but there was no trade-off between safety and efficiency because they are driven by different xylem anatomical traits that are under different phylogenetic control.

## Teaser

We used a common garden experiment to assess the mechanisms underlying embolism resistance and hydraulic conductivity of conifer species. Pit size and sealing explain embolism resistance, while hydraulic conductivity is only related to wall thickness.

## Introduction

Drought triggers tree mortality across the globe, because it can lead to impairment of water transport and eventually hydraulic failure, desiccation, and tree death (Urli *et al.*, 2013; Choat *et al.*, 2018). The risk of hydraulic failure depends on the hydraulic architecture of a tree, which consists of a series of water-transporting conduits in the xylem and tiny pits that connect these neighbouring conduits. Gymnosperm trees (i.e., conifers) are considered on average more drought resistant than Angiosperm trees due to specific anatomical features. Conifers possess many narrow and short conduits called tracheids that allow for slow but safe water transport, and for resistance against drought- and freezing-induced embolism (Davis *et al.*, 1999). These tracheids are interconnected through pits, which usually possess a torus that acts as a safety valve and seals the pit, and can thus avoid drought-induced embolism (Pittermann *et al.*, 2005). These unique anatomical properties of conifers may contribute to their success and dominance in harsh habitats, either in cold boreal and mixed temperate forests, or in dry Mediterranean forests (Augusto *et al.*, 2014). Despite these unique anatomical features, conifer species still vary substantially in their vulnerability to drought (Larter *et al.*, 2017).

### The function of pits for hydraulic strategies

**Fig. 1.**
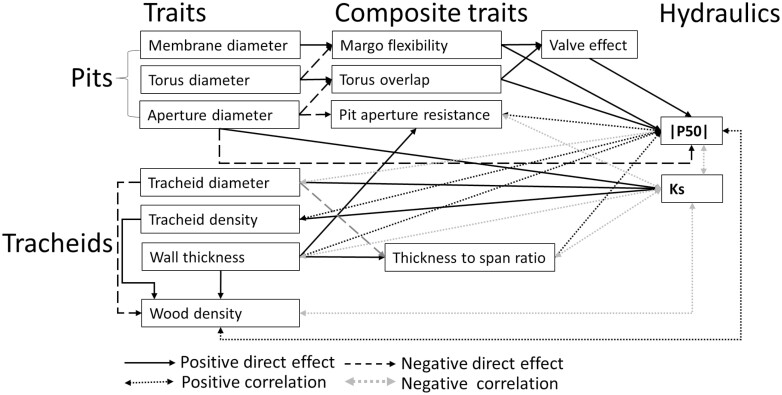
A conceptual diagram visualizing our hypothesis for how pit and tracheid traits jointly determine species differences in embolism resistance (|P50|) and hydraulic conductivity (Ks). Our trait list is shown in Table 1.

Embolism can move by air seeding from one conduit to an adjacent conduit, and thus distribute air within the xylem and block water flow in more xylem conduits (Cochard, 2006; Guan *et al.*, 2021). The seeding of air bubbles into the conduits is thought to occur through the interconduit pits (Zimmermann, 1983). Normally, these pits in the xylem of conifer species consist of an aperture through which water can flow from one tracheid to the other, and an impermeable torus that can seal the pit under negative pressure (Bauch *et al.*, 1972; Hacke *et al.*, 2004). The torus is surrounded by a flexible margo that allows the torus to move and seal the pit. The margo also acts as a membrane, through which water can flow when the pit is not sealed (Hacke *et al.*, 2004; Sperry *et al.*, 2006). This pit structure plays an important role in the hydraulic efficiency, which is often quantified by Ks (xylem-specific hydraulic conductivity), and embolism resistance, which is often quantified by P50 (the water potential at which 50% of the conductivity is lost) (Delzon *et al.*, 2010) (Fig. 1). A larger pit aperture reduces the resistance to water flow and thus increases hydraulic efficiency, but may indirectly reduce torus overlap (Hacke and Jansen, 2009; Jansen and McAdam, 2019), facilitating torus slippage or movement and therefore mass flow of gas that causes embolism (Fig. 1). Embolism resistance may increase with torus overlap, when there is a large overlap between torus and pit aperture, and with a flexible margo, as high flexibility may better seal a pore (Fig. 1). Considerable uncertainty remains about the role of margo flexibility, as some studies found that margo flexibility does not determine embolism resistance (Hacke *et al.*, 2004; Bouche *et al*., 2014, 2015). In combination, the torus overlap and margo flexibility result in a valve effect (i.e., effective sealing) of the pit (Fig. 1), which is the best predictor of embolism resistance across 40 conifer tree species (Sperry *et al.*, 2006; Delzon *et al.*, 2010).

### The function of tracheids for hydraulic strategies

Embolism resistance and hydraulic efficiency are also related to tracheid traits. Conifer xylem mainly consists of tracheids and small areas of parenchyma. As there is only limited space to pack the tracheids, there is a trade-off between tracheid density and tracheid diameter. Theoretical specific hydraulic conductivity increases linearly with tracheid density, as each additional tracheid contributes equally to water transport, but increases with the tracheid diameter to the power 4, as in wide conduits there is relatively less friction between water and the cell wall (Tyree and Ewers, 1991; Sterck *et al.*, 2008). In addition, tracheid wall thickness can also indirectly affect hydraulic conductivity, because thick cell walls reduce the water flow due to a reduced lumen diameter (Sperry *et al.*, 2006) and increase the water flow path through pits and thus the pit hydraulic resistance (Hacke *et al.*, 2004; Bouche *et al.*, 2014). A greater wall thickness to tracheid diameter ratio reinforces the mechanical resistance against tracheid implosion due to increased negative pressure during drought (Jansen *et al.*, 2009), and the related traits such as large cell wall thickness and small lumen area are positively associated with embolism resistance (Bouche *et al.*, 2014; Fig. 1). It should be noted that tracheid implosion, however, has rarely been observed in nature, probably because the conduits are mechanically overbuilt. Despite the mechanistic basis of these links between the tracheid traits and hydraulic performance, a five decades old common garden experiment enables us to fully assess the underlying mechanism in hydraulic performance across a broad range of conifer species within the same environment.

### Trade-off between hydraulic efficiency and safety

Conifer species with small pits and tracheids may thus have a high resistance to drought-induced embolism, which is also known as hydraulic ‘safety’, but this comes at the cost of a reduced water flow and water transport capacity, which is also known as hydraulic ‘efficiency’ (Liu *et al.*, 2019). Yet, several studies observed only a weak or even no trade-off between hydraulic safety and hydraulic efficiency (Pittermann *et al.*, 2006; Larter *et al.*, 2017), possibly because some of the postulated mechanisms are causing stronger constraints on these processes than others. The current consensus seems to be that embolism resistance in gymnosperms is mainly determined by pit sealing (Delzon *et al.*, 2010; Bouche *et al.*, 2014), whereas hydraulic efficiency is thought to be mainly determined by tracheid size and density (Sterck *et al.*, 2008).

In this study, we compare the hydraulic conductivity and embolism resistance of 28 dominant conifer species from the Northern hemisphere that cover a broad range in phylogenetic and ecology diversity. They come from different habitats including cold habitats where conifers are thought to have narrow tracheids to avoid freezing-induced embolism (Leslie *et al.*, 2012; Zanne *et al.*, 2014), and xeric habitats with high resistance to embolism. While earlier studies evaluated embolism resistance of conifer species by comparing species sampled from different areas (Hacke and Jansen, 2009; Delzon *et al.*, 2010; Bouche *et al.*, 2014; Losso *et al.*, 2018), our common garden approach allows us to focus on inherent species differences not confounded by acclimation to different environmental conditions. In addition, we will use phylogenetic analysis to show phylogenetic effects on trait variation. We address the following three questions and corresponding hypotheses (Fig. 1; see conceptual diagram).

(i) How is xylem resistance to embolism determined by tracheid and pit traits? We expect that embolism resistance increases when the likelihood of air seeding is reduced, because of either smaller pit dimensions (due to smaller tracheid dimensions; see Hacke *et al.*, 2004) or more efficient pit sealing properties (i.e., large torus overlap, margo flexibility, and valve effect). We expect species with high embolism resistance to have mechanically enforced tracheids (high thickness to span ratio) and tissues (high wood density) to protect tracheid against collapse under extreme negative pressure. (ii) How is hydraulic conductivity determined by tracheid and pit traits? We expect hydraulic conductivity to increase with tracheid width and pit aperture, because they reduce the friction between water and the cell wall and therefore facilitate water flow. (iii) To what extent are the hydraulic traits of conifer species phylogenetically controlled? We expect that embolism resistance and the related pit traits, and hydraulic conductivity and the related tracheid traits, exhibit strong phylogenetic signals because radiation in conifer species only followed after adaptation to drought or water availability (Larter *et al.*, 2017).

## Materials and methods

### Study site and species selection

We conducted our study in the Schovenhorst Estate (52.25N, 5.63E) in Putten, the Netherlands. Within this region, the mean annual temperature is 10.1 °C, the maximum annual temperature is 13.5 °C, the minimum annual temperature is 6.0 °C, the mean annual rainfall is 830mm, and the elevation is ~30 m above sea level. Soils are derived from post-glacial loamy sand deposits, forming well-drained and acidic (pH ~4) podzolic soils of low fertility (Cornelissen *et al.*, 2012; van der Wal *et al.*, 2016).

The research was carried out in a common garden experiment, where >30 conifer species from the Northern hemisphere were introduced and planted in 1966 in monospecific stands (Willinge Gratama-Oudemans, 1992). Such a common garden experiment allows comparison of the performance of different species under similar climatic and soil conditions, thus correcting for potentially confounding phenotypic responses to environmental variation. We selected 28 conifer species with sufficient replicate trees available for this study (Supplementary Table S1).

These species were selected if at least five healthy individuals reached the forest canopy, and were thus fully exposed and able to achieve full growth potential. Species had an average stem diameter at breast height of 35.8cm (range 5.0–86.3cm). We sampled one 38–45cm long branch for each of the five individuals per species. These branches were fully exposed to reduce phenotypic variation and collected at ~6 m (5–7 m) above the forest floor. After cutting, the main stem segment of the branch was stripped from leaves and side branches, and wrapped in wet paper and sealed in a plastic bag to avoid transpiration and embolism. Samples were sent to the GENOBOIS platform (a high-throughput phenotyping platform for physiological traits, CaviPlace lab. University of Bordeaux. Pessac. France http://sylvain-delzon.com/caviplace/) and stored in a fridge at 3–5 °C prior to measurements.

We measured the vulnerability curve for these five branches, and for three out of those five branches, pit and tracheid properties were measured. Another group of branches was collected in June 2019 to measure wood density based on five individuals per species.

### Vulnerability curves

**Fig. 2.**
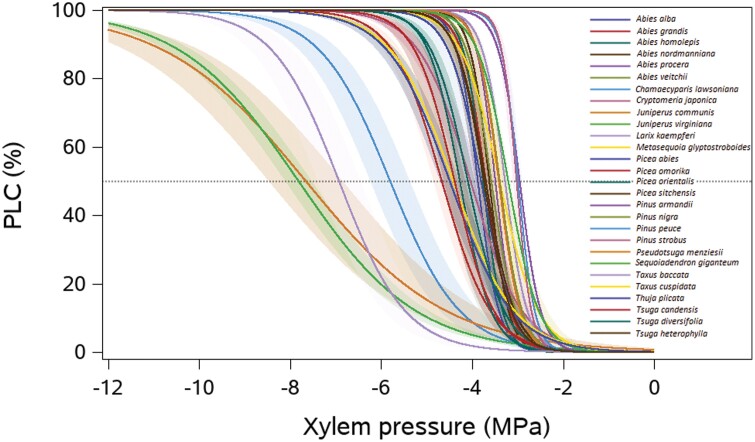
The vulnerability curves for 28 conifer species in this study. PLC indicates the percentage loss of hydraulic conductivity. Different colours indicate different species.


Five branches per species were collected on adult trees to characterize xylem resistance to embolism using the standard ‘Cavitron’ method, where a centrifugal force was used to establish negative pressure in the xylem to provoke drought-induced embolism (Cochard *et al.*, 2005). Centrifugal force was created using a 27cm wide custom-built honeycomb aluminium rotor (DGmeca, Gradignan, France) mounted on a high-speed centrifuge (Sorvall RC5C Plus Refrigerated Centrifuge, USA) at 20 °C , thus controlling the temperature-dependent viscosity of water and gas solubility of the perfusion liquid (Wang *et al.*, 2014; Schenk *et al.*, 2016). All measurements were done at the PHENOBOIS platform at INRAE-University of Bordeaux. Prior to measurements, all samples were re-cut underwater to a length of 27cm and the bark was removed using a razor blade. A solution of 10mM KCl and 1mM CaCl_2_ in ultrapure deionized water was used as the reference ionic solution. The rotor was first spun at low xylem pressure (−0.8MPa) and the rotation speed of the centrifuge was then gradually increased by −0.3MPa or −0.8MPa, depending on the species, to expose samples to lower xylem pressures. After exposing the sample at the required speed for 2min, hydraulic conductance (k in m^2^ MPa^–1^ s^–1^) was measured three times per speed step. The xylem-specific hydraulic conductivity (Ks in kg m^-1^ MPa^–1^ s^–1^, i.e., hydraulic efficiency) was estimated when xylem pressure (P in MPa) was close to zero, which was calculated by dividing the maximum hydraulic conductance (k_max_ in m^2^ MPa^–1^ s^–1^) by sample length and sapwood area (Larter *et al.*, 2017). Vulnerability curves show the percentage loss of hydraulic conductance in response to increased negative xylem pressure (Fig. 2). The percentage loss of hydraulic conductance (PLC) at each pressure was subsequently calculated as:

$$\begin{array}{l} PLC=100\left(1k/k_{max}\right) \end{array}$$ (1)

Then a sigmoid function was performed to fit the curves as follows (Fig. 2):


$$\begin{array}{l} PLC=100/\left\{1+exp\left[S/25\times \left(PP50\right)\right]\right\} \end{array}$$ (2)

where S (% MPa^–1^) is the slope at the inflection point in the vulnerability curve, and P50 (MPa) is the xylem pressure when 50% of hydraulic conductance is lost (Delzon *et al.*, 2010). All values were averaged at the species level. The absolute value of P50 (|P50|) is then used for further analysis and is referred to as embolism resistance.

### Xylem anatomy measurements

**Fig. 3.**
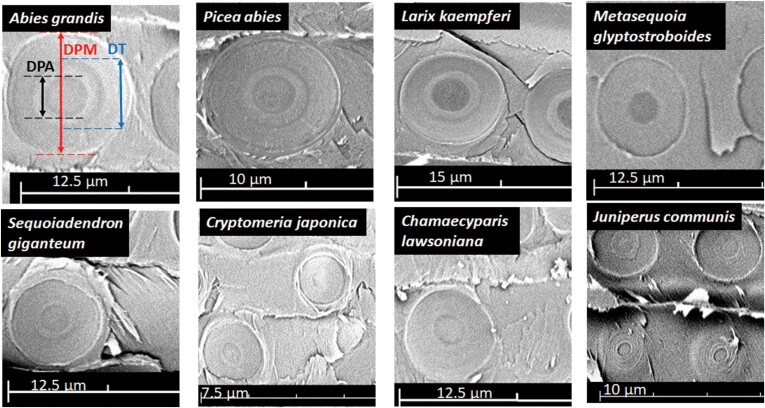
Scanning electron microscopy images of pit structure in the earlywood of eight conifers species illustrating the variation in pit size across study species. Different pit traits are indicated with different colours. DPM, pit membrane diameter (red colour); DT, torus diameter (blue colour); DPA, pit aperture diameter (black colour).


After measurements of the vulnerability curves, two 2–3cm segments for three individual branches per species were reserved for measuring pit and tracheid anatomical traits (Table 1). Traits related to pit size and pit sealing were measured in the earlywood using scanning electron microscopy at the PHENOBOIS platform (PhenomG2 pro; FEI, the Netherlands). For this purpose, 10 pit membranes were measured per selected segment. Those segment samples were first dried for 24h in an oven at 65 °C and then cut with a razor blade in a radial direction. Samples were subsequently coated with a thin layer of gold using a sputter coater (108 Auto; Cressington, UK) for 40s at 20 mA (Bouche *et al.*, 2014). The following pit size measurements were estimated in earlywood using ImageJ v. 1.52a.: pit aperture diameter (DPA), torus diameter (DT), and pit membrane diameter (DPM) (Fig. 3). We focused on pit characteristics of earlywood because earlywood has the strongest effect on the embolism resistance of plants (Domec and Gartner, 2002). To assess the sealing function of the torus for embolism resistance, three anatomical traits were calculated according to Delzon *et al.* (2010): margo flexibility (MF), torus overlap (TO), and valve effect (VE). MF was estimated as a proxy for flexibility based on the length of the margo stretch, although it does not account for the mechanical properties of the margo. MF, TO, and VE were calculated as follows (Delzon *et al.*, 2010):

**Table 1.** Overview of traits, the abbreviations. and units as used and measured for trees of 28 conifer species in the Netherlands

Classification	Trait name	Abbreviation	Units
Pit	Pit membrane diameter	DPM	µm
	Torus diameter	DT	µm
	Pit aperture diameter	DPA	µm
	Torus overlap	TO	–
	Margo flexibility	MF	–
	Valve effect	VE	–
	Pit aperture resistance	R_PA_	MPa s m^–3^
Tracheid	Hydraulic diameter	Dh	µm
	Tracheid density	TD	mm^–2^
	Tracheid diameter (earlywood)	D_E	µm
	Tracheid diameter (latewood)	D_L	µm
	Tracheid density (earlywood)	TD_E	mm^–2^
	Tracheid density (latewood)	TD_L	mm^–2^
	Wall thickness	Tw	µm
	Wall thickness (earlywood)	Tw_E	µm
	Wall thickness (latewood)	Tw_L	µm
	Thickness to span ratio	TSR	µm µm^–1^
	Thickness to span ratio (earlywood)	TSR_E	µm µm^–1^
	Thickness to span ratio (latewood)	TSR_L	µm µm^–1^
	Wood density	WD	g cm^-3^
Hydraulics	Embolism resistance	|P50|	MPa
	Xylem specific hydraulic conductivity	Ks	Kg m^–1^ s^–1^ MPa^–1^

$$\begin{array}{l} TO=\left(DT-DPA\right)/DT \end{array}$$ (3)

$$\begin{array}{l} MF=\left(DPM-DT\right)/DPM \end{array}$$ (4)

$$\begin{array}{l} VE=TO\times MF \end{array}$$ (5)

To evaluate how hydraulic resistance affects hydraulic conductivity and embolism resistance, pit aperture resistance (R_PA_) was calculated following Pittermann *et al.* (2010) and Bouche *et al.* (2014):


$$\begin{array}{l} R_{PA}=[128T_{pa}t/{\left(pDPA\right)}^{4}+24t/DPA^{3}] \end{array}$$ (6)

where t is the viscosity of the water (0.001 Pa s at 20 °C) and T_pa_ is the thickness of a single pit border calculated from wall thickness (T_pa_=81%×2Tw; Domec *et al.*, 2008).

To explore how tracheid traits affect hydraulic conductivity and embolism resistance, tracheid diameter, tracheid density, and cell wall thickness were subsequently measured on a complete radial segment on cross-sections of three branches per species. For the cross-sections, we used the same three branches that were used for the measurement of the vulnerability curve. These cross-sections were cut with a thickness between 15 μm and 30 μm. The sections were stained with Astra blue and safranin for 3–5min to ensure a better differentiation of the tissues (lignified tissues acquire a red colour and non-lignified tissues acquire a blue colour). They were sequentially washed 1–3 times with distilled water, and 50, 75, 96, and 100% ethanol. Finally, Roti®-Mount was used to fix the samples and make images of transverse sections at ×10 or ×20 magnification with a Leica DM 2500 camera microscope and LAS V 3.8 software. To get a complete image of the radial section, we used the photo stitching software PTGui v.9.2.0 to stitch those pictures (Supplementary Fig. S1). Pictures of the tracheid diameter (in μm), tracheid density (i.e., tracheid number per area in mm^–2^), and cell wall thickness (in μm) were measured using ImageJ v.1.52a. Tracheid diameter (D), tracheid density (TD), and cell wall thickness (Tw) were averaged on a minimum of 200 tracheids per sample. The thickness to span ratio (TSR) was calculated as the square of the ratio of double wall thickness to lumen diameter (Hacke *et al.*, 2001). To reduce the measurement errors caused by the irregular shapes of tracheids, we measured the area of the tracheid. From this, we mathematically derived the tracheid diameter, assuming a circular shape. For four tracheid traits (tracheid diameter, tracheid density, wall thickness, and thickness to span ratio), we calculated average tracheid characteristics in three ways: (i) for the complete radial section, which should best reflect whole-branch functioning; (ii) for the earlywood, as this may contribute most to hydraulic conductivity and conductivity loss during embolism; and (iii) for the latewood. Hydraulic diameter (D_h_) was calculated based on the whole radial section, as it best reflects hydraulic conductivity of the whole branch, and as it weighs for differences in tracheid diameter between earlywood and latewood by using the Hagen–Poiseuille equation. A principal component analysis (PCA) shows that, across species, tracheid characteristics of earlywood, latewood, and the whole radial section were strongly aligned (Supplementary Fig. S2). Additional statistical analysis based on earlywood tracheid values and latewood tracheid values (Supplementary Figs S3, S4) gave very similar results to those for the average tracheid values, indicating that the results are robust. For further analysis, we therefore used the average tracheid characteristics across the whole radial section as this should best reflect whole-branch functioning. The TSR and D_h_ were calculated as (Sterck *et al.*, 2008; Poorter *et al.*, 2010):


$$\begin{array}{l} TSR={\left(2\times Tw/D\right)}^{2} \end{array}$$ (7)

$$\begin{array}{l} D_{h}=\sqrt[{4}]{\frac{1}{n}\underset{i=1}{\overset{n}{\sum }}\;Di^{4}} \end{array}$$ (8)

where *Di* is the *i*th conduit of *n* measured tracheids.

### Wood density

A section of 10cm length was selected to measure wood density (WD, in g cm^–3^). Wood fresh volume was measured using the water displacement method (Poorter *et al.*, 2010). Wood samples without bark were dried in an oven at 70 °C to obtain constant dry weight after 48h, and wood density was calculated as the dry mass to volume ratio per sample.

### Data analysis

To explore the significant differences of P50 and Ks among 28 conifer species, data were cleaned by removing outliers and transformed to meet the normality and homogeneity assumptions of ANOVA, then a post-hoc Turkey (HSD) test was performed.

To evaluate how traits are associated with each other, a PCA was first carried out, using the 14 traits (Table 1) of 28 species. We then used regression analyses and Pearson correlations to explore the pairwise relationships between tracheid, pit, and hydraulic traits. To evaluate whether the present-day cross-species correlation results from repeated independent adaptations during evolution, we also calculated correlations based on phylogenetically independent contrasts (PICs), using the R package ‘ape’ (Paradis *et al.*, 2004; Poorter *et al.*, 2010).

To assess how pit and tracheid traits affect embolism resistance and hydraulic conductivity, a multiple regression was performed using the dredge function in the R package ‘MuMIn’ (Barton and Barton, 2015). A dredge function allows evaluation of the importance of all possible driving variables underlying variation in P50 and Ks, avoiding the statistical bias of dropping or entering variables in a backward or forward regression approach. To avoid problems with multicollinearity, only traits were selected with a variance inflation factor (VIF) <5 (Comont *et al.*, 2012; Gould *et al.*, 2016). We focused on traits that could be mechanistically linked to Ks and P50. The potential regression model for Ks included D_h_, TD, and R_PA_, and the potential regression model for P50 included DPA, MF, and VE. The best model was the model with the lowest Akaike information criterion (AICc). ΔAICc for *i*th model was calculated as the difference between the AICc for the *i*th model and the best model. A set of models with ΔAICc <2 was selected (Araujo *et al.*, 2019).

To explore the cause and effect pathways of pit traits on embolism resistance and tracheid traits on hydraulic conductivity, we performed structural equation models (SEMs). The structure of SEMs was based on our conceptual diagram (Fig. 1). To compare the effect sizes, all data were standardized prior to analysis by subtracting the mean from the trait values and dividing it by the SD. The model was accepted when the *P*-value of the χ^2^ statistic was >0.05 (Poorter *et al.*, 2017). SEMs were built in the R package ‘lavaan’ (Rosseel, 2012).

**Fig. 4.**
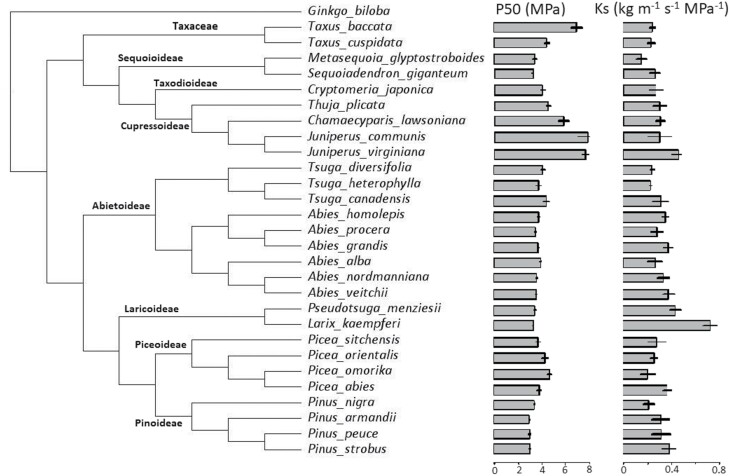
Phylogenetic tree of the 28 conifer species with embolism resistance (|P50|) and hydraulic conductivity (Ks) in this study, based on the molecular phylogeny from Zanne *et al.* (2014). *Ginkgo biloba* is selected as the outlier and reference.

To evaluate to what extent hydraulic features are phylogenetically controlled, Blomberg’s *K* (Blomberg *et al.*, 2003) values were calculated with the R ‘phytools’ package (Revell, 2012). *K* values close to 0 indicate that traits show no phylogenetic signal (i.e., close relatives differ more in their trait values than distant relatives because of the independent evolution of traits). *K* values close to 1 indicate a significant phylogenetic signal in the evolution of traits under Brownian motion, a random motion model; and *K*>1 indicates that the trait is strongly phylogenetically conserved (Kamilar and Cooper, 2013). Differences between large phylogenetic groups based on the subfamily (i.e., Pinoideae, Piceoideae, Laricoideae, Abietoideae, Sequoioideae, Cupressoideae, Taxodioideae, and Taxaceae; Fig. 4) were evaluated using one-way ANOVA with Tukey’s (HSD) post-hoc tests. As there was no subfamily within Taxaceae, Taxaceae was used here for the analysis. All data were implemented in the R statistical environment (R Core Team, 2020).

## Results

### Interspecific variation for P50 and Ks

The embolism resistance (P50) varied significantly across species (ANOVA, *F*_27,110_=34.2, *P*<0.001), and ranged from –2.96MPa for *Pinus armandii* to –7.82MPa for *Juniperus virginiana* (Figs 2, 4; Supplementary Fig. S3A). P50 also varied strongly amongst families (ANOVA, *F*_2,135_=22.4, *P*<0.001) and amongst species within the Pinaceae (ANOVA, *F*_18,74_=19.1, *P*<0.001), Cupressaceae (ANOVA, *F*_6,28_=39.5, *P*<0.001), and Taxaceae (*F*_1,8_=31.2, *P*<0.001). The hydraulic conductivity (Ks) also varied significantly across species (ANOVA, *F*_27,112_=4.2, *P*<0.001), and ranged from 0.15kg^–1^ m s^–1^ MPa^–1^ for *Picea omorika* to 0.68kg^–1^ m s^–1^ MPa^–1^ for *Larix kaempferi* (Fig. 4; Supplementary Fig. S3B). Ks also differed significantly amongst species within the Pinaceae (ANOVA, *F*_18,72_=4.9, *P*<0.001) and Cupressaceae (ANOVA, *F*_6,32_=3.0, *P*=0.02). However, there were no differences for Ks amongst families (ANOVA, *F*_2,137_=2.5, *P*=0.09) and amongst species within Taxaceae (*F*_1,8_=0.2, *P*=0.68).

### Trait associations

**Fig. 5.**
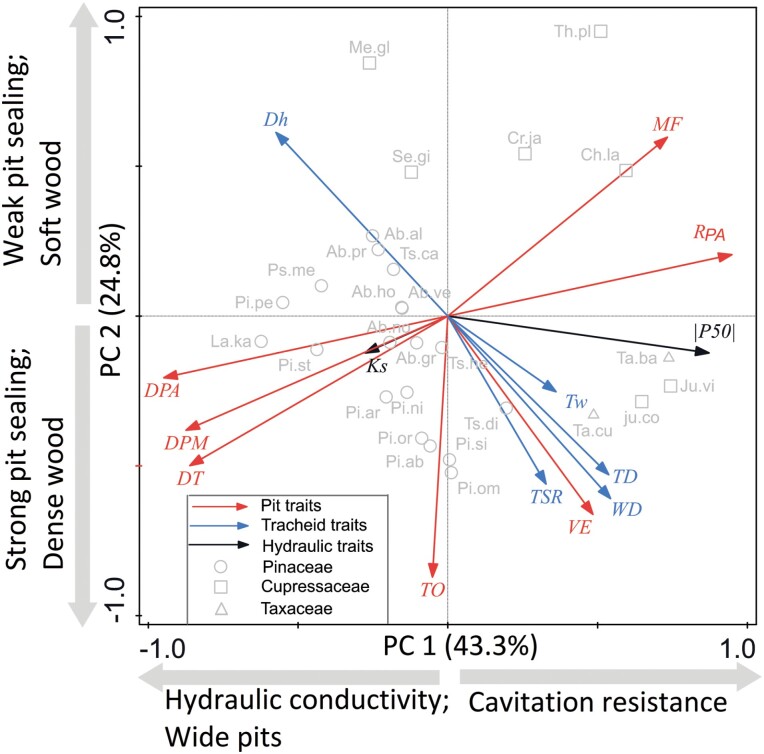
Principal components analysis (PCA) of multivariate trait associations across 28 conifer species. The first two PCA axes and the loadings of 14 traits are shown. Different trait groups are indicated with different coloured arrows (pit traits=red, tracheid traits=blue, hydraulic traits=black). Different families (Cupresssaceae, Pinaceae, Taxaceae) are indicated by different symbols. For trait abbreviations (in italic black), see Table 1; for species abbreviations (in black), see Supplementary Table S1.

The first two PCA axes explained 68.3% of the variation and we found two spectra of traits (Fig. 5). The first axis reflects a trade-off between hydraulic safety and hydraulic efficiency, with high embolism resistance, pit aperture resistance, and margo flexibility for mainly Cupressaceae and Taxaceae species on the right side of the axis, and high hydraulic conductivity and wide pit dimensions for multiple Pinaceae species on the left side. The second axis reflects a trade-off between tracheid number (at the bottom) and tracheid size (at the top), dense (bottom) versus porous wood (high D_h_, top), and strong pit sealing (high TO and VE). Therefore, three spectra can be defined along the second axis, namely size–number spectrum for tracheids, ranging from large numbers of tracheids at the bottom to wide tracheids at the top, a toughness spectrum ranging from hardwood with dense wood (WD) and strong cell wall reinforcement (TSR) at the bottom to softwood at the top, and a pit sealing spectrum ranging from strong sealing at the bottom to weak sealing at the top. These hydraulic spectra reflect an old split between embolism-resistant Cupressaceae and Taxaceae with high margo flexibility (MF) to the right, and conductive Pinaceae with wide pits and potentially fast growth to the left. Within these two groups, species vary along a gradient from tough and expensive tissues (high WD) to soft, porous, and cheap tissues (high D_h_).

### Effects of pit and tracheid characteristics on embolism resistance

**Fig. 6.**
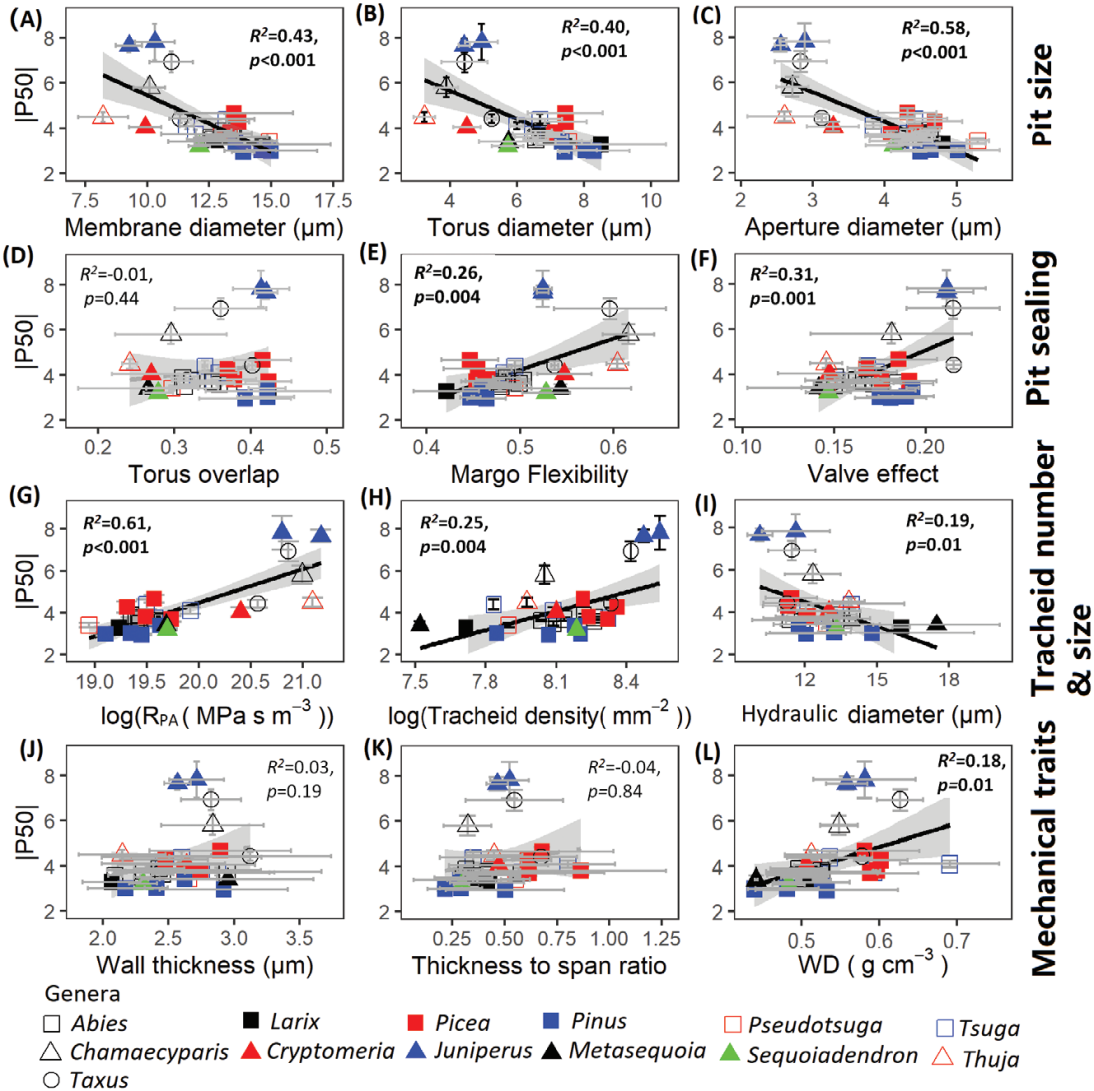
Bivariate relationships between embolism resistance (P50) and underlying properties for 28 conifer tree species. The traits are grouped in rows according to their function: pit size, pit sealing, tracheid traits, and mechanical traits. For trait abbreviations, see Table 1. Bivariate error bars (±SE of the mean), regression lines and 95% confidence intervals (grey), coefficients of determination (*R*^2^), and *P*-value are shown.

First, we used pairwise and multiple regression to statistically explore what traits (best) predict P50 and Ks. All pit and tracheid traits were correlated with embolism resistance. Pairwise regressions between |P50| and anatomical traits showed that pit size (i.e., pit aperture diameter, pit membrane diameter, and torus diameter) and tracheid size (i.e., hydraulic diameter) were negatively related to embolism resistance, whereas tracheid density, margo flexibility, and mechanical resistance (R_PA_, pit aperture resistance; WD, wood density) were positively related to embolism resistance (Fig. 6). Multiple regression indicated that embolism resistance increased significantly with valve effect (VE) and decreased with pit aperture size (DPA) (Table 2).

**Table 2.** The results of a multimodel comparison showing how embolism resistance (|P50|) and hydraulic conductivity (Ks) depend on pit and tracheid traits

model	DPA	VE	D_h_	TD	Intercept	df	logLik	AICc	Weight	R^2^_adj_	P
|P50|=DPA+MF+VE											
1	**–0.64**	**0.31**			3.89×10^–16^	4	–23.59	56.90	0.62	0.65	<0.001
Ks=D_h_+TD+R_PA_											
1					–1.05×10^–16^						
2				–0.20	–1.63×10^–16^	3	–38.63	84.30	0.21		
3			0.16		–3.72×10^–17^	3	–38.87	84.70	0.16		
Avg			0.03	–0.05	–1.01×10^–16^						
Imp			0.21	0.27	1.00						
*P*			0.77	0.69	1.00						

Only the best models (ΔAIC<2) were included, and averaged (in the case of Ks). Bold indicates significant coefficients.

Values indicate regression coefficients of the selected variables in the model. Per model, degrees of freedom (df), the log likelihood (logLik), corrected Akaike information criterion (AICc), and the AICc weight are given. Models are selected based on ΔAIC<2. The average model was calculated based on the selected models. The average coefficients (Avg), relative importance (Imp), and significances (*P*) are shown. Relative importance of the predictor variables is calculated as the sum of the Akaike weights over the best selected models. D_h_, hydraulic diameter; TD, tracheid density; DPA, pit aperture diameter; MF, margo flexibility; VE, valve effect; R_PA_, pit aperture resistance.

**Fig. 7.**
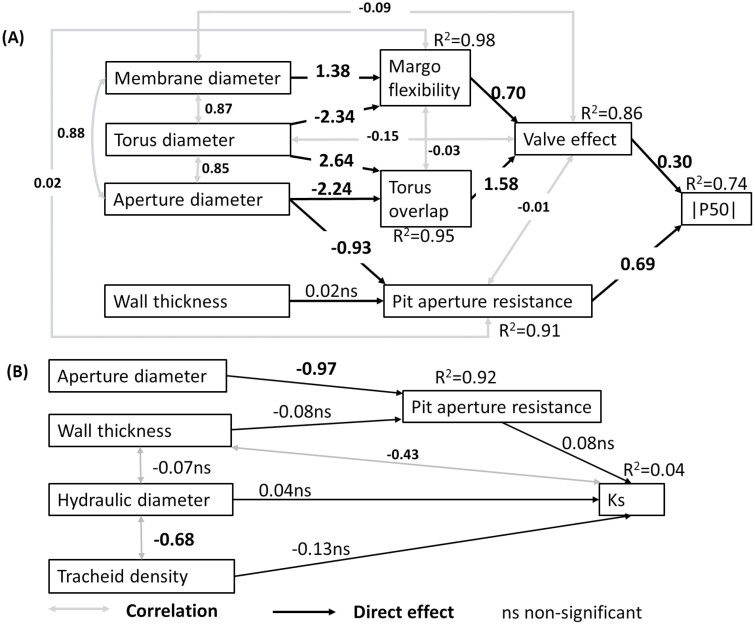
Structural equation models for the effects of pit and tracheid traits on hydraulics for 28 conifer species: (A) embolism resistance (|P50|) (χ^2^=27.17, df=18, *P*=0.08), (B) hydraulic conductivity (Ks) (χ^2^=13.71, df=7, *P*=0.06). Significant standardized coefficients are shown in bold, and ns means non-significant. The standardized coefficients, significant effects. and total effects of traits on |P50|or Ks can be found in Supplementary Table S1.

Second, we tested our conceptual path model (Fig. 1) of how traits are organized hierarchically and affect each other through a chain of cause–effect relationships. As expected, valve effect and pit aperture resistance directly and positively affected |P50| (Fig. 7A). Margo flexibility and torus overlap had significantly indirect and positive effects on |P50| through their positive effects on the valve effect (Fig. 7A; Supplementary Table S2). Similar results were obtained when we used tracheid trait values of earlywood (Supplementary Fig. S4A) or latewood (Supplementary Fig. S4B).

### Effects of pit and tracheid characteristics on hydraulic conductivity

**Fig. 8.**
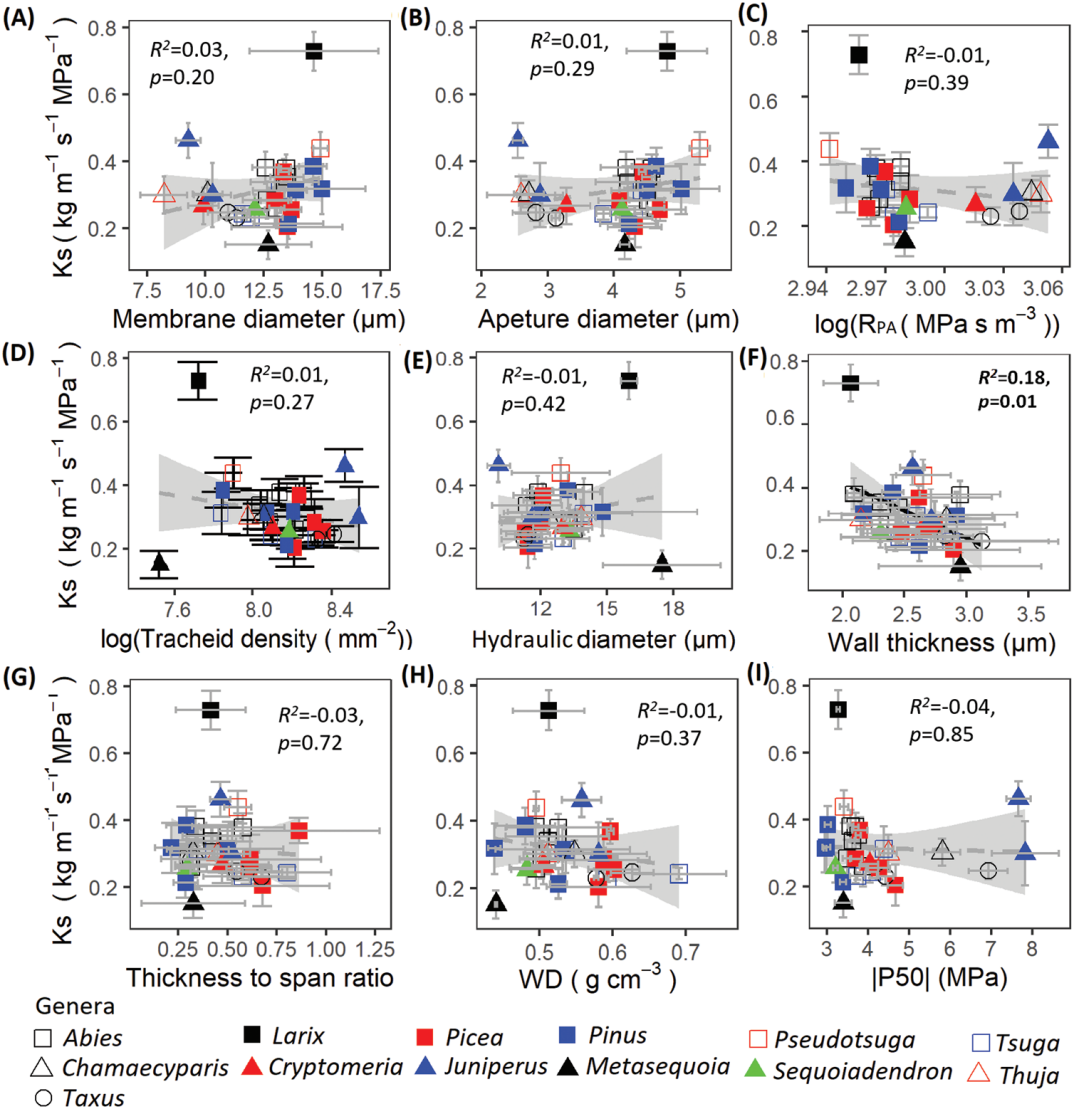
Bivariate relationships between maximum specific hydraulic conductivity (Ks) and underlying properties (A–H) for 28 conifer tree species. The trade-off between Ks and embolism resistance (|P50|) is shown (I). The traits are grouped in rows according to their function; pit traits (A–C), tracheid traits (D, E), and mechanical traits (F–H). For trait abbreviations, see Table 1. Bivariate error bars (±SE of the mean), regression lines, and 95% confidence intervals (grey), coefficients of determination (*R*^2^), and *P*-value are shown.

The pairwise regression between Ks, pit, and tracheid traits showed that Ks was only significantly and negatively related to wall thickness (Fig. 8; Table 3). Multiple regression of Ks on tracheid density, D_h_, and pit aperture resistance showed that none of these traits could significantly explain Ks (Table 2; Supplementary Table S3). The structural equation model further proved that only conduit wall thickness was negatively correlated with Ks (Fig. 7B), which was the same when we constrained the analyses based on tracheid traits of earlywood (Supplementary Fig. S4C). Surprisingly, there was no correlation between hydraulic conductivity and embolism resistance (Fig. 8I).

### Phylogenetic correlations and phylogenetic signals

We used Blomberg’s *K* metrics to evaluate whether P50, Ks, and related pit and tracheid traits of conifer species are phylogenetically conserved. The hydraulic traits P50 and Ks, and most traits related to pit size and pit sealing showed significant phylogenetic signals (Table 4). To explore how phylogenetic groups differed from each other, the 28 species were classified into eight groups based on subfamily (Table 4; Fig. 4). Cupressoideae differed significantly from Laricoideae, Pinoideae, and Sequoioideae, but not from the Taxaceae. The Cupressoideae were compared with Abietoideae, Piceoideae, and Laricoideae, and were found to be more drought tolerant and embolism resistant (high |P50|) because of a smaller pit size (DPM, DT, DPA), and they had higher pit aperture resistance and margo flexibility (Table 4).

**Table 4.** Differences in hydraulics and underlying traits among eight phylogenetic groups (Piceoideae, Pinoideae, Laricoideae, Abietoideae, Cupressoideae, Taxodioideae, Sequoioideae, and Taxaceae; see Fig. 4

Traits		Phylogenetic test		One-way ANOVA		Mean value							
		Blomberg’s *K*-value *P-*value		F_7, 20_	P	Piceoideae	Pinoideae	Laricoideae	Abietoideae	Cupressoideae	Taxodioideae	Sequoioideae	Taxaceae
Pit size	Pit membrane diameter (DPM) (µm)	**1.28**	**0.001**	25.48	**<0.001**	13.37 DE	14.25 E	14.78 E	12.81 CD	9.48 A	9.93 AB	12.39 BCD	11.15 ABC
	Torus diameter (DT) (µm)	**1.82**	**0.001**	34.91	**<0.001**	7.28 DE	7.78 E	8.03 E	6.60 CD	4.11 A	4.49 AB	5.73 BC	4.84 AB
	Aperture diameter (DPA) (µm)	**1.62**	**0.001**	19.39	**<0.001**	4.38 CD	4.59 A	5.05 E	4.35 CD	2.69 A	3.28 ABC	4.14 BCD	2.98 AB
Pit sealing	Torus overlap (TO)	**0.60**	**0.002**	3.15	**0.02**	0.40 AB	0.41 B	0.36 AB	0.34 AB	0.34 AB	0.27 A	0.27 A	0.38 AB
	Margo flexibility (MF)	**0.89**	**0.001**	11.62	**<0.001**	0.45 A	0.45 A	0.46 A	0.48 A	0.57 B	0.55 AB	0.54 AB	0.57 B
	Valve effect (VE)	**0.56**	**0.001**	4.78	**0.003**	0.18 AB	0.18 AB	0.16 A	0.16 A	0.19 AB	0.15 A	0.14 A	0.22 B
Tracheid size	Hydraulic diameter (D_h_) (µm)	0.42	0.07	2.37	0.06	11.73 A	12.95 A	14.46 A	12.72 A	11.97 A	12.99 A	15.40 A	11.36 A
	Tracheid density (TD) (mm^–2^)	0.34	0.13	2.36	0.06	3.96×10^3^ A	3.24×10^3^ A	2.47×10^3^ A	3.46×10^3^ A	3.98×10^3^ A	3.29×10^3^ A	2.72×10^3^ A	4.35×10^3^ A
Mechanics	Wall thickness (Tw) (µm)	0.27	0.38	0.89	0.53	2.68 A	2.53 A	2.36 A	2.50 A	2.57 A	2.64 A	2.63 A	2.97 A
	Thickness to span ratio (TSR)	**0.43**	**0.02**	**3.26**	**0.02**	0.69 B	0.32 A	0.48 AB	0.46 AB	0.44 AB	0.47 AB	0.31 AB	0.61 AB
	Wood density (WD) (g cm^–3^)	**0.62**	**0.002**	3.23	**0.02**	0.59 B	0.49 AB	0.50 AB	0.54 AB	0.55 AB	0.51 AB	0.46 AB	0.60 B
	Pit aperture resistance (R_PA_) (MPa s m^–3^)	**1.04**	**0.001**	18.57	**<0.001**	3.06×10^8^ BC	2.66×10^8^ AB	1.97×10^8^ A	3.12×10^8^ BC	1.35×10^9^ E	7.27×10^8^ CDE	3.57×10^8^ BCD	1.00×10^9^ DE
Hydraulics Embolism resistance (|P50|) (MPa)		**0.53**	**0.03**	13.69	**<0.001**	4.11 BC	3.09 A	3.35 AB	3.81 AB	6.44 D	4.04 ABC	3.30 AB	5.67 CD
Maximum specific hydraulic		**0.49**	**0.04**	3.67	**0.01**	0.28 A	0.31 A	0.58 B	0.31 A	0.34 AB	0.27 A	0.20 A	0.24 A
conductivity (Ks) (kg m^–1^ s^–1^ MPa^–1^)													

Phylogenetic test for traits and one-way ANOVA with Tukey’s (HSD) post-hoc test. Groups that are significantly different (*P*<0.05) are indicated with different letters. Bold represents significant values.

Out of the 91 possible pairwise trait correlations, 27 correlations were significant, both across species and using phylogenetic correlations (Table 3). Eleven significant cross-species correlations became non-significant when using phylogenetic correlations. These correlations mainly referred to the relationship between P50 and tracheid traits (i.e., D_h_ and TD) and Ks versus Tw. Two correlations that were not significant across species became significant using a phylogenetic correlation; WD was negatively related to pit aperture diameter (DPA) and resistance (R_PA_) (Table 3).

**Table 3.** Correlations between traits related to hydraulics (white columns), tracheid traits (grey columns), pit traits (blue columns), and wood mechanical traits (green column) for 28 conifer species

Trait	Hydraulics		Tracheid traits				Pit traits							WD
	P50	Ks	D_h_	TD	Tw	TSR	DPM	DT	DPA	TO	MF	VE	R_PA_	
P50		0.11	–0.20	0.22	–0.02	–0.05	–0.13	–0.34	**–0.52**	0.19	**0.44**	**0.49**	**0.53**	**0.47**
Ks	–0.05		–0.18	–0.03	–0.28	0.06	0.00	–0.07	–0.11	0.09	0.13	0.18	0.11	0.02
Dh	**–0.51**	0.03		**–0.52**	0.10	–0.24	0.32	0.20	**0.46**	–0.37	0.003	**–0.42**	**–0.46**	**–0.53**
TD	**0.53**	–0.09	**–0.75**		–0.08	0.27	–0.30	–0.04	–0.17	0.23	–0.30	0.08	0.17	**0.53**
Tw	0.29	**–0.48**	–0.22	0.14		**0.59**	0.28	0.30	0.11	0.36	–0.16	0.31	–0.09	0.20
TSR	0.20	–0.11	–0.33	0.34	**0.47**		–0.13	0.06	–0.14	0.36	–0.24	0.26	0.15	**0.61**
DPM	**–0.69**	0.17	0.27	–0.29	–0.09	–0.14		**0.83**	**0.78**	0.35	–0.19	0.29	**–0.77**	–0.32
DT	**–0.67**	0.22	0.19	–0.19	–0.14	–0.03	**0.96**		**0.85**	**0.55**	**–0.70**	0.19	**–0.84**	–0.27
DPA	**–0.80**	0.14	**0.38**	–0.35	–0.21	–0.08	**0.93**	**0.93**		0.03	**–0.50**	–0.28	**–0.99**	**–0.51**
TO	0.10	0.27	**–0.43**	0.36	0.11	0.13	**0.38**	**0.50**	0.14		**–0.51**	**0.83**	–0.02	0.33
MF	**0.56**	–0.26	–0.09	0.04	0.20	–0.12	**–0.78**	**–0.92**	**–0.79**	**–0.60**		0.06	**0.50**	0.07
VE	**0.52**	0.13	**–0.61**	**0.49**	0.29	0.08	–0.09	–0.04	**–0.39**	**0.81**	–0.02		0.28	**0.44**
R_PA_	**0.80**	–0.14	**–0.38**	0.35	0.22	0.08	**–0.93**	**–0.93**	**–1.00**	–0.14	**0.79**	**0.40**		**0.51**
WD	**0.52**	–0.13	**–0.66**	**0.53**	0.37	**0.72**	–0.27	–0.18	–0.33	0.35	0.02	**0.47**	0.33	

Pairwise Pearson correlations (below the diagonal) and phylogenetically independent contrast correlations (above the diagonal) are shown. Bold values represent significant correlations at *P*<0.05. For trait abbreviations, see Table 1

## Discussion

We show how 28 conifer species differed in P50 and Ks, and how those differences were explained from the underlying anatomical pit and tracheid traits. We found that the valve effect and pit aperture resistance determined the xylem embolism resistance. Hydraulic conductivity was, surprisingly, not explained by pit and tracheid size, but was only negatively related to wall thickness. Embolism resistance and its underlying anatomical traits were under stronger phylogenetic control than hydraulic efficiency. Below we will discuss how pit and tracheid traits affect embolism resistance and hydraulic conductivity, and how pit traits, tracheid traits, and hydraulics are phylogenetically controlled.

### Embolism resistance: the function of pits and tracheids

We hypothesized that embolism resistance would increase with high pit aperture resistance resultant from a small pit aperture and thick cell wall, and with strong pit sealing properties and particularly with a strong valve effect as driven by large torus overlap and high margo flexibility. We indeed found support for this hypothesis. As expected, small pit dimensions (as characterized by small pit aperture diameter, pit membrane diameter, and torus diameter) were indeed associated with a high embolism resistance (Fig. 6A–C, I). The structural equation model implies that the effects of small pits, in combination with thick cell walls, particularly increase the pit aperture resistance, which increases the embolism resistance (Fig. 7A; Supplementary Fig. S4A). This is in line with the idea that a small pit size increases embolism resistance due to a relatively increased torus overlap, because the torus size remains fairly constant, whereas a reduction in pit aperture leads to a stronger increase in torus overlap (Bouche *et al.*, 2014; Jansen and McAdam, 2019), but adds a significant role for the cell wall thickness of earlywood (Supplementary Fig. S4A). The structural equation model also showed that the valve effect positively affected embolism resistance (Fig. 7A), which is thought to be the best integrator of pit sealing properties (Delzon *et al.*, 2010). Both torus overlap and margo flexibility had a significant indirect effect via the valve effect on embolism resistance (Fig. 7A; Supplementary Table S2). These results are at least partially supported by the bivariate relationships with embolism resistance (Fig. 6): the valve effect showed the strongest relationship (Fig. 6F), followed by margo flexibility (Fig. 6E), but remarkably torus overlap did not have any bivariate trend with |P50| (Fig. 6D) despite a strong contribution to variation in the valve effect (Fig. 7). These results are in line with the aspiration hypothesis, which suggests that air seeding occurs when the torus cannot perfectly seal the aperture against the pit border (the inner wall of the pit membrane) (Pittermann *et al.*, 2010). The positive trend between margo flexibility and embolism resistance (Fig. 6E) is also in line with Hacke *et al.* (2004), implying that a flexible margo enables the torus to seal the aperture perfectly against the pit border (Delzon *et al.*, 2010). Our results nevertheless most strongly support the aspiration hypothesis in the sense that a stronger valve effect is the most direct way of sealing the pit aperture with a direct positive effect on embolism resistance (Figs 6F, 7).

The torus capillary seeding hypothesis is an alternative hypothesis for linking pit traits to embolism resistance, and suggests that air seeding occurs at the pores of the torus (Jansen *et al.*, 2009; Bouche *et al.*, 2014). In a study of 13 conifer species, it was observed that embolism resistance was indeed determined by the size of torus pores, as air seeding occurs firstly at the largest pore (Jansen *et al.*, 2012). Because we did not measure the pores in the punctured tori, we cannot test for this mechanism. Finally, we found that embolism resistance increased with tracheid density (Fig. 6H), which is consistent with other studies (Jacobsen *et al.*, 2016, see Supplementary Table S1; Larter *et al.*, 2017). Perhaps a larger amount of smaller tracheids provides extra safety because there are more functional tracheids left to take over the role of embolized tracheids. Alternatively, more tracheids per area indicate more cell wall and less lumen per area, leading to stronger limitations for gas diffusion and embolism.

### Why is hydraulic conductivity not explained by pit and tracheid traits?

Hydraulic conductivity (Ks) is often found to increase with pit and tracheid size (Pittermann *et al.*, 2006; Sperry *et al.*, 2006; Woodruff *et al.*, 2008; Hacke and Jansen, 2009), as water transport through wide structures reduces friction between water and plant cells, and facilitates water flow (Roskilly *et al.*, 2019). In contrast to these predictions, neither pit aperture diameter nor hydraulic diameter was related to hydraulic conductivity in our study (Figs 7B, 8B, E) (cf. Larter *et al.*, 2017). We found that hydraulic conductivity was only significantly and negatively related to tracheid cell wall thickness (Figs 7B, 8F). Thick cell walls may affect hydraulic conductivity in two ways: they may increase the hydraulic pathlength within the pits, and therefore pit aperture resistance, or they may reduce the lumen area available for fluid flow (Hacke *et al.*, 2004). Yet, neither of these two mechanisms is likely to have played a role in our study. First, cell wall thickness contributed little to pit aperture resistance (which is mostly determined by pit aperture diameter), and pit aperture resistance did not have a significant effect on Ks (Figs 7B, 8C; Supplementary Fig. S4C). Second, although there is indeed a negative correlation between wall thickness and hydraulic diameter (*r*= –0.22, Table 3), this is not significant. In addition, in our multiple regression analysis (Table 2) and structural equation model (Fig 7B), we checked for independent effects of cell wall thickness and D_h_ on Ks, and D_h_ never had a significant effect. Hence, cell wall thickness is associated with Ks, but for unknown reasons.

It should be said that the observed range of hydraulic conductivity is rather small (ranging from 0.15kg m^–1^ s^–1^ MPa^–1^ to 0.46kg m^–1^ s^–1^ MPa^–1^ across species, excluding the outlier *Larix* (Fig. 8) which may explain our inability to detect significant relationships between Ks and putative tracheid traits. Alternatively, it could be that hydraulic conductivity is determined by other hydraulic bottlenecks, such as the sizes of pores in the margo (Schulte *et al.*, 2015), the overlapping tracheid tips due to bent tracheids, or tracheid length since that determines the flow path or affects the end wall conductivity which contributes nearly to 64% of total resistivity in tracheids (Pittermann *et al.*, 2006; Sperry *et al.*, 2006), but those properties were not considered here.

### Trait associations and trade-offs

It was expected that there would be a trade-off between hydraulic efficiency and hydraulic safety, because large tracheids and pits potentially increase the hydraulic conductivity but come at the cost of reduced embolism resistance to air seeding. We did not find, however, any relationship between hydraulic conductivity and embolism resistance (Fig. 8I) as previously reported by several studies (Gleason *et al.*, 2016). We used a PCA to identify major hydraulic spectra and trade-offs (Fig. 5). The first PCA axis represents the major axis of hydraulic trait variation, showing a trade-off between hydraulic safety (i.e., high embolism resistance and pit resistance) and pit dimensions (i.e., pit size). Species with high embolism resistance need small pits to enhance resistance to air seeding (Sano, 2016), which we also observed in the bivariate trait associations (Figs 6A–C; Table 3). The second PCA axis shows three spectra, namely a size–number spectrum for tracheids, a toughness spectrum, and a pit sealing spectrum (Fig. 5). These three spectra indicate that species with strong pit sealing capacity need to invest resources in tough structures such as dense wood, thick cell wall reinforcement, and dense tracheids to prevent embolism, which comes at the cost of water transport through tracheids. Structural equation models proved that hydraulic conductivity was only related to tracheid wall thickness (Fig. 7B), whereas embolism resistance was determined by pit size and pit sealing properties through a valve effect (Fig. 7A). The major variation in embolism resistance and hydraulic efficiency is driven by different sets of traits, which explains why hydraulic efficiency and hydraulic safety were disconnected. In that sense, our study is in line with other recent studies (Willson *et al.*, 2008; Larter *et al.*, 2017), which fail to show a trade-off between these potentially competing hydraulic functions, and does provide a possible explanation for this.

### Phylogenetic signal and trait evolution

P50 varied significantly across species, with *Pinus* species being the most drought intolerant and *Juniperus* species being the most drought tolerant (Fig. 4). Blomberg’s *K* values showed that embolism resistance and its underlying pit traits (pit size and pit sealing) were phylogenetically conserved, indicating that they are the result of old phylogenetic splits. The ancestral traits are largely maintained because insufficient time has passed since the evolutionary divergence (Ackerly, 2003) (Table 4). Most of the significant PICs were among P50 and pit traits, indicating that increased embolism resistance has evolved in combination with decreased pit aperture size, and increased pit aperture resistance and valve effect (Table 3). Traits that reflect mechanical reinforcement (i.e., Tw, TSR, and R_PA_) and, hence, material construction costs (Poorter *et al.*, 2018) also showed a significant phylogenetic signal (cf. Chave *et al.*, 2006).

Pit size traits were more conserved than pit sealing traits (MF, TO, and VE), probably because pit size is closely related to—and the result of—cell size, which may be more difficult to change in response to different environmental conditions during evolution (David-Schwartz *et al.*, 2016). Pit sealing traits may be easier to modify, as they result from two underlying pit size traits (Fig. 1); a small increase in one pit size trait combined with a small decrease in another may therefore lead to larger changes and more flexibility in pit sealing traits.

The strong phylogenetic control of pit size traits therefore explains the relatively strong phylogenetic control of embolism resistance and drought adaptation. Cupressoideae and Taxaceae were the most embolism-resistant (i.e., high |P50|) phylogenetic groups because of their small pit sizes, strong pit sealing property (MF), and high pit aperture resistance (R_PA_). Instead, the fast-growing and light-demanding Pinoideae (Webb and Scanga, 2001) and Laricoideae (Dobrovolný *et al.*, 2013) had larger pit dimensions (DPM, DT, and DPA) and weaker pit sealing (MF) than the slow-growing Cupressoideae. We conclude that closely joined evolution of high embolism resistance with small pit size, a higher valve effect, and pit aperture resistance has enabled conifer species to be very resistant to drought (Pittermann *et al.*, 2010).

In contrast, hydraulic conductivity and its underlying tracheid traits (tracheid size, tracheid number, and cell wall thickness) were under much weaker phylogenetic control, suggesting that hydraulic conductivity traits may have allowed species to radiate into different habitats (Panek, 1996). Weak phylogenetic signals in hydraulic conductivity have also been found in other studies on broadleaf species (Liang *et al.*, 2019) and conifer species (Corcuera *et al.*, 2011). Phenotypic plasticity may play a vital role in the variation of hydraulic traits in relation to water availability (Liang *et al.*, 2019), but such variation was not considered in our common garden study. Possibly, our common garden approach led to smaller hydraulic conductivity differences between the conifer species than when comparing such species as acclimated in their natural, contrasting, habitats.

### Conclusions

We compared the hydraulics of 28 conifer species grown under standardized conditions in a common garden experiment. Pit sealing properties, tracheid size, and tracheid numbers indeed affect the embolism resistance of conifer species. Anatomical stem traits (pit size, margo flexibility, torus overlap, and valve effect) of conifers are phylogenetically conserved, and strongly control species differences in embolism resistance. Unexpectedly, hydraulic conductivity was weakly phylogenetically controlled, and negatively related to tracheid wall thickness, rather than being related to hydraulic diameter or pit aperture size. Future studies could explore the role of tracheid length and margo pores in hydraulic conductivity. In sum, conifer species differ greatly in embolism resistance and the underlying traits, and in hydraulic conductivity, and they may therefore differ strongly in their climatic distribution and drought responses to climate change.

## Supplementary data

The following supplementary data are available at *JXB* online.

Fig. S1. An example of a radial subsection from the cross-section for the measurements of *Tsuga heterophylla*.

Fig. S2. Principal components analysis (PCA) of multivariate trait associations across 28 conifer species.

Fig. S3. Boxplot of P50 (the xylem pressure when 50% of hydraulic conductance is lost) and Ks (maximum specific hydraulic conductivity) across 28 conifer species.

Fig. S4. Structural equation models for the effects of pit traits and tracheid traits of earlywood and latewood on the embolism resistance (|P50|) and hydraulic conductivity (Ks) for 28 conifer species

Table S1. Overview of species, abbreviations, family, subfamily, and genera of 28 conifer species in the Netherlands.

Table S2. Results of the structural equation models for the effects of pit and tracheid traits on embolism resistance (|P50) and hydraulic conductivity (Ks) shown in Fig. 7.

Table S3. Results of a multi-model comparison showing how hydraulic conductivity (Ks) depends on the tracheid traits of earlywood and latewood.

erab449_suppl_Supplementary_Figures_S1-S4_Tables_S1-S3

## Data Availability

The average species trait data are available upon reasonable request from the authors.

## References

[CIT0001] Ackerly DD. 2003. Community assembly, niche conservatism, and adaptive evolution in changing environments. International Journal of Plant Sciences 164, S165–S184.

[CIT0002] Araujo FDC , Tng DYP, Apgaua DMG, Morel JD, Pereira DGS, Santos PF, Santos RMd. 2019. Flooding regime drives tree community structure in Neotropical dry forests. Journal of Vegetation Science 30, 1195–1205.

[CIT0003] Augusto L , Davies TJ, Delzon S, De Schrijver A. 2014. The enigma of the rise of angiosperms: can we untie the knot? Ecology Letters 17, 1326–1338. 24975818 10.1111/ele.12323

[CIT0004] Barton K , Barton MK. 2015. Package ‘MuMIn’. Version 1, 18. https://cran.r-project.org/web/packages/MuMIn/MuMIn.pdf.

[CIT0005] Bauch J , Liese W, Schultze R. 1972. The morphological variability of the bordered pit membranes in gymnosperms. Wood Science and Technology 6, 165–184.

[CIT0006] Blomberg SP , Garland T Jr, Ives AR. 2003. Testing for phylogenetic signal in comparative data: behavioral traits are more labile. Evolution 57, 717–745. 12778543 10.1111/j.0014-3820.2003.tb00285.x

[CIT0007] Bouche PS , Jansen S, Cochard H, Burlett R, Capdeville G, Delzon S. 2015. Embolism resistance of conifer roots can be accurately measured with the flow-centrifuge method.Journal of Plant Hydraulics 2, e002.

[CIT0008] Bouche PS , Larter M, Domec JC, Burlett R, Gasson P, Jansen S, Delzon S. 2014. A broad survey of hydraulic and mechanical safety in the xylem of conifers. Journal of Experimental Botany 65, 4419–4431. 24916072 10.1093/jxb/eru218 PMC4112641

[CIT0009] Chave J , Muller-Landau HC, Baker TR, Easdale TA, Steege HT, Webb CO. 2006. Regional and phylogenetic variation of wood density across 2456 neotropical tree species. Ecological Applications 16, 2356–2367. 17205910 10.1890/1051-0761(2006)016[2356:rapvow]2.0.co;2

[CIT0010] Choat B , Brodribb TJ, Brodersen CR, Duursma RA, López R, Medlyn BE. 2018. Triggers of tree mortality under drought. Nature 558, 531–539. 29950621 10.1038/s41586-018-0240-x

[CIT0011] Cochard H. 2006. Cavitation in trees. Comptes Rendus Physique 7, 1018–1026.

[CIT0012] Cochard H , Damour G, Bodet C, Tharwat I, Poirier M, Améglio T. 2005. Evaluation of a new centrifuge technique for rapid generation of xylem vulnerability curves. Physiologia Plantarum 124, 410–418.

[CIT0013] Comont RF , Roy HE, Lewis OT, Harrington R, Shortall CR, Purse BV. 2012. Using biological traits to explain ladybird distribution patterns. Journal of Biogeography 39, 1772–1781.

[CIT0014] Corcuera L , Cochard H, Gil-Pelegrin E, Notivol E. 2011. Phenotypic plasticity in mesic populations of *Pinus pinaster* improves resistance to xylem embolism (P 50) under severe drought. Trees 25, 1033–1042.

[CIT0015] Cornelissen JH , Sass-Klaassen U, Poorter L, et al. 2012. Controls on coarse wood decay in temperate tree species: birth of the LOGLIFE experiment. Ambio 41 Suppl 3, 231–245. 22864697 10.1007/s13280-012-0304-3 PMC3535053

[CIT0016] David-Schwartz R , Paudel I, Mizrachi M, Delzon S, Cochard H, Lukyanov V, Badel E, Capdeville G, Shklar G, Cohen S. 2016. Indirect evidence for genetic differentiation in vulnerability to embolism in *Pinus halepensis*. Frontiers in Plant Science 7, 768. 27313594 10.3389/fpls.2016.00768 PMC4889591

[CIT0017] Davis SD , Sperry JS, Hacke UG. 1999. The relationship between xylem conduit diameter and cavitation caused by freezing. American Journal of Botany 86, 1367–1372. 10523278

[CIT0018] Delzon S , Douthe C, Sala A, Cochard H. 2010. Mechanism of water-stress induced cavitation in conifers: bordered pit structure and function support the hypothesis of seal capillary-seeding. Plant, Cell & Environment 33, 2101–2111. 10.1111/j.1365-3040.2010.02208.x PMC3003904 20636490

[CIT0019] Dobrovolný L , Štěrba T, Kodeš J. 2013. Effect of stand edge on the natural regeneration of spruce, beech and douglas-fir. Acta Universitatis Agriculturae et Silviculturae Mendelianae Brunensis 60, 49–56.

[CIT0020] Domec JC , Gartner BL. 2002. How do water transport and water storage differ in coniferous earlywood and latewood? Journal of Experimental Botany 53, 2369–2379. 12432029 10.1093/jxb/erf100

[CIT0021] Domec JC , Lachenbruch B, Meinzer FC, Woodruff DR, Warren JM, McCulloh KA. 2008. Maximum height in a conifer is associated with conflicting requirements for xylem design. Proceedings of the National Academy of Sciences, USA 105, 12069–12074. 10.1073/pnas.0710418105 PMC2575339 18695232

[CIT0022] Gleason SM , Westoby M, Jansen S, et al. 2016. Weak tradeoff between xylem safety and xylem-specific hydraulic efficiency across the world’s woody plant species. New Phytologist 209, 123–136. 10.1111/nph.13646 26378984

[CIT0023] Gould IJ , Quinton JN, Weigelt A, De Deyn GB, Bardgett RD. 2016. Plant diversity and root traits benefit physical properties key to soil function in grasslands. Ecology Letters 19, 1140–1149. 27459206 10.1111/ele.12652 PMC4988498

[CIT0024] Guan X , Pereira L, McAdam SAM, Cao KF, Jansen S. 2021. No gas source, no problem: proximity to pre-existing embolism and segmentation affect embolism spreading in angiosperm xylem by gas diffusion. Plant, Cell & Environment 44, 1329–1345. 10.1111/pce.14016 33529382

[CIT0025] Hacke UG , Jansen S. 2009. Embolism resistance of three boreal conifer species varies with pit structure. New Phytologist 182, 675–686. 10.1111/j.1469-8137.2009.02783.x 19309447

[CIT0026] Hacke UG , Sperry JS, Pittermann J. 2004. Analysis of circular bordered pit function II. Gymnosperm tracheids with torus–margo pit membranes. American Journal of Botany 91, 386–400. 21653394 10.3732/ajb.91.3.386

[CIT0027] Hacke UG , Sperry JS, Pockman WT, Davis SD, McCulloh KA. 2001. Trends in wood density and structure are linked to prevention of xylem implosion by negative pressure. Oecologia 126, 457–461. 28547229 10.1007/s004420100628

[CIT0028] Jacobsen AL , Tobin MF, Toschi HS, Percolla MI, Pratt RB. 2016. Structural determinants of increased susceptibility to dehydration-induced cavitation in post-fire resprouting chaparral shrubs. Plant, Cell & Environment 39, 2473–2485. 10.1111/pce.12802 27423060

[CIT0029] Jansen S , Choat B, Pletsers A. 2009. Morphological variation of intervessel pit membranes and implications to xylem function in angiosperms. American Journal of Botany 96, 409–419. 21628196 10.3732/ajb.0800248

[CIT0030] Jansen S , Lamy JB, Burlett R, Cochard H, Gasson P, Delzon S. 2012. Plasmodesmatal pores in the torus of bordered pit membranes affect cavitation resistance of conifer xylem. Plant, Cell & Environment 35, 1109–1120. 10.1111/j.1365-3040.2011.02476.x 22220551

[CIT0031] Jansen S , McAdam S. 2019. Pits with aspiration explain life expectancy of a conifer species. Proceedings of the National Academy of Sciences, USA 116, 14794–14796. 10.1073/pnas.1909866116 PMC6660727 31289227

[CIT0032] Kamilar JM , Cooper N. 2013. Phylogenetic signal in primate behaviour, ecology and life history. Philosophical Transactions of the Royal Society B: Biological Sciences 368, 20120341. 10.1098/rstb.2012.0341 PMC3638444 23569289

[CIT0033] Larter M , Pfautsch S, Domec JC, Trueba S, Nagalingum N, Delzon S. 2017. Aridity drove the evolution of extreme embolism resistance and the radiation ofconifer genus *Callitris*. New Phytologist 215, 97–112. 10.1111/nph.14545 28378882

[CIT0034] Leslie AB , Beaulieu JM, Rai HS, Crane PR, Donoghue MJ, Mathews S. 2012. Hemisphere-scale differences in conifer evolutionary dynamics. Proceedings of the National Academy of Sciences, USA 109, 16217–16221. 10.1073/pnas.1213621109 PMC3479534 22988083

[CIT0035] Liang X , He P, Liu H, Zhu S, Uyehara IK, Hou H, Wu G, Zhang H, You Z, Xiao Y. 2019. Precipitation has dominant influences on the variation of plant hydraulics of the native *Castanopsis fargesii* (Fagaceae) in subtropical China. Agricultural and Forest Meteorology 271, 83–91.

[CIT0036] Liu H , Gleason SM, Hao G, Hua L, He P, Goldstein G, Ye Q. 2019. Hydraulic traits are coordinated with maximum plant height at the global scale. Science Advances 5, eaav1332. 30788435 10.1126/sciadv.aav1332 PMC6374111

[CIT0037] Losso A , Anfodillo T, Ganthaler A, Kofler W, Markl Y, Nardini A, Oberhuber W, Purin G, Mayr S. 2018. Robustness of xylem properties in conifers: analyses of tracheid and pit dimensions along elevational transects. Tree Physiology 38, 212–222. 29309674 10.1093/treephys/tpx168

[CIT0038] Panek JA. 1996. Correlations between stable carbon-isotope abundance and hydraulic conductivity in Douglas-fir across a climate gradient in Oregon, USA. Tree Physiology 16, 747–755. 14871681 10.1093/treephys/16.9.747

[CIT0039] Paradis E , Claude J, Strimmer K. 2004. APE: Analyses of Phylogenetics and Evolution in R language. Bioinformatics 20, 289–290. 14734327 10.1093/bioinformatics/btg412

[CIT0040] Pittermann J , Choat B, Jansen S, Stuart SA, Lynn L, Dawson TE. 2010. The relationships between xylem safety and hydraulic efficiency in the Cupressaceae: the evolution of pit membrane form and function. Plant Physiology 153, 1919–1931. 20551212 10.1104/pp.110.158824 PMC2923884

[CIT0041] Pittermann J , Sperry JS, Hacke UG, Wheeler JK, Sikkema EH. 2005. Torus–margo pits help conifers compete with angiosperms. Science 310, 1924. 16373568 10.1126/science.1120479

[CIT0042] Pittermann J , Sperry JS, Hacke UG, Wheeler JK, Sikkema EH. 2006. Inter-tracheid pitting and the hydraulic efficiency of conifer wood: the role of tracheid allometry and cavitation protection. American Journal of Botany 93, 1265–1273. 21642190 10.3732/ajb.93.9.1265

[CIT0043] Poorter L , Castilho CV, Schietti J, Oliveira RS, Costa FRC. 2018. Can traits predict individual growth performance? A test in a hyperdiverse tropical forest. New Phytologist 219, 109–121. 10.1111/nph.15206 PMC6001574 29774944

[CIT0044] Poorter L , McDonald I, Alarcón A, Fichtler E, Licona JC, Peña-Claros M, Sterck F, Villegas Z, Sass-Klaassen U. 2010. The importance of wood traits and hydraulic conductance for the performance and life history strategies of 42 rainforest tree species. New Phytologist 185, 481–492. 10.1111/j.1469-8137.2009.03092.x 19925555

[CIT0045] Poorter L , van der Sande MT, Arets EJ, Ascarrunz N, Enquist BJ, Finegan B, Licona JC, Martínez-Ramos M, Mazzei L, Meave JA. 2017. Biodiversity and climate determine the functioning of Neotropical forests. Global Ecology and Biogeography 26, 1423–1434.

[CIT0046] R Core Team . 2020. R: a language and environment for statistical computing. Vienna, Austria: R Foundation for Statistical Computing.

[CIT0047] Revell LJ. 2012. phytools: an R package for phylogenetic comparative biology (and other things). Methods in Ecology and Evolution 3, 217–223.

[CIT0048] Roskilly B , Keeling E, Hood S, Giuggiola A, Sala A. 2019. Conflicting functional effects of xylem pit structure relate to the growth–longevity trade-off in a conifer species. Proceedings of the National Academy of Sciences, USA 116, 15282–15287. 10.1073/pnas.1900734116 PMC6660759 31209057

[CIT0049] Rosseel Y. 2012. Lavaan: an R package for structural equation modeling and more. Version 0.5- 12 (BETA). Journal of Statistical Software 48, 1–36.

[CIT0050] Sano Y. 2016. Bordered pit structure and cavitation resistance in woody plants. In: Kim YS, Funada R, Singh AP, eds. Secondary xylem biology. Origins, functions, and applications. Elsevier, 113–130.

[CIT0051] Schenk HJ , Espino S, Visser A, Esser BK. 2016. Dissolved atmospheric gas in xylem sap measured with membrane inlet mass spectrometry. Plant, Cell & Environment 39, 944–950. 10.1111/pce.12678 26868162

[CIT0052] Schulte PJ , Hacke UG, Schoonmaker AL. 2015. Pit membrane structure is highly variable and accounts for a major resistance to water flow through tracheid pits in stems and roots of two boreal conifer species. New Phytologist 208, 102–113. 10.1111/nph.13437 25944400

[CIT0053] Sperry JS , Hacke UG, Pittermann J. 2006. Size and function in conifer tracheids and angiosperm vessels. American Journal of Botany 93, 1490–1500. 21642096 10.3732/ajb.93.10.1490

[CIT0054] Sterck FJ , Zweifel R, Sass-Klaassen U, Chowdhury Q. 2008. Persisting soil drought reduces leaf specific conductivity in Scots pine (*Pinus sylvestris*) and pubescent oak (*Quercus pubescens*). Tree Physiology 28, 529–536. 18244940 10.1093/treephys/28.4.529

[CIT0055] Tyree MT , Ewers FW. 1991. The hydraulic architecture of trees and other woody plants. New Phytologist 119, 345–360.

[CIT0056] Urli M , Porté AJ, Cochard H, Guengant Y, Burlett R, Delzon S. 2013. Xylem embolism threshold for catastrophic hydraulic failure in angiosperm trees. Tree Physiology 33, 672–683. 23658197 10.1093/treephys/tpt030

[CIT0057] van der Wal A , Klein Gunnewiek PJ, Cornelissen JHC, Crowther TW, de Boer W. 2016. Patterns of natural fungal community assembly during initial decay of coniferous and broadleaf tree logs. Ecosphere 7, e01393.

[CIT0058] Wang Y , Burlett R, Feng F, Tyree M. 2014. Improved precision of hydraulic conductance measurements using a Cochard rotor in two different centrifuges. Journal of Plant Hydraulics 1, e0007.

[CIT0059] Webb SL , Scanga SE. 2001. Windstorm disturbance without patch dynamics: twelve years of change in a Minnesota forest. Ecology 82, 893–897.

[CIT0060] Willinge Gratama-Oudemans JJ. 1992. The arboretum of Schovenhorst, Putten, in the Netherlands. Arboricultural Journal 16, 197–205.

[CIT0061] Willson CJ , Manos PS, Jackson RB. 2008. Hydraulic traits are influenced by phylogenetic history in the drought-resistant, invasive genus *Juniperus* (Cupressaceae). American Journal of Botany 95, 299–314. 21632355 10.3732/ajb.95.3.299

[CIT0062] Woodruff DR , Meinzer FC, Lachenbruch B. 2008. Height-related trends in leaf xylem anatomy and shoot hydraulic characteristics in a tall conifer: safety versus efficiency in water transport. New Phytologist 180, 90–99. 10.1111/j.1469-8137.2008.02551.x 18631290

[CIT0063] Zanne AE , Tank DC, Cornwell WK, et al. 2014. Three keys to the radiation of angiosperms into freezing environments. Nature 506, 89–92. 24362564 10.1038/nature12872

[CIT0064] Zimmermann M. 1983. Xylem structure and the ascent of sap. Berlin: Springer. 10.1126/science.222.4623.500-a 17746198

