# Supplementary material for: Pit and tracheid anatomy explain hydraulic safety but not hydraulic efficiency of 28 conifer species
Source: J Exp Bot. 2021 Oct 9;73(3):1033–48. doi: 10.1093/jxb/erab449 (PMC8793876; doi:10.1093/jxb/erab449)
Supplement: erab449_suppl_Supplementary_Figures_S1-S4_Tables_S1-S3 [file erab449_suppl_supplementary_figures_s1-s4_tables_s1-s3.pdf]

Supplementary data

Article title: **Pits and tracheids explain the hydraulic safety- but not the hydraulic efficiency of 28 conifer species**

The following Supplementary data is available for this article:

Fig. S1 An example of a radial subsection (blue) from the cross-section for the measurements of *Tsuga heterophylla*. The tracheid diameter and wall thickness for earlywood (green) and latewood (black) were measured based on a radial subsection (blue).

Fig. S2 Boxplot of P50 (the xylem pressure when 50% of hydraulic conductance is lost) and Ks (xylem specific hydraulic conductivity) across 28 conifer species (N=3-7 trees/species). Species with different letters are significantly different (Tukey HSD post hoc test, $P<0.05$). Error bars (± standard error of the mean value) are shown.

Fig. S3 Principal components analysis (PCA) of multivariate trait associations across 28 conifer species. The first two PCA axes and the loadings of 14 traits are shown. Different trait groups are indicated with different colored arrows for pit traits (red), earlywood traits (blue), latewood traits (green), average wood traits of early and latewood (purple), and hydraulic traits (black). Different families (Cupressaceae, Pinaceae, Taxaceae) are indicated by different symbols. For trait abbreviations (in Italics), see Table 1, for species abbreviations (in grey) see Table S1.

Fig. S4 Structural equation models for the effects of pit traits and tracheid traits of earlywood (a, c) and latewood (b, d) on the cavitation resistance (|P50|) and hydraulic conductivity (Ks) for 28 conifer species: (a) earlywood and |P50| (χ^2=23.38, df=17, P=0.053), (b) latewood and (|P50|) (χ^2=20.34, df=18, P=0.31), (c) earlywood and Ks (χ^2=19.18, df=7, P=0.01), (d) latewood and Ks (χ^2=15.76, df=7, P=0.03). The pit characteristics were all for earlywood, except pit aperture resistance could vary between early and latewood for P50; and tracheid diameter, density, and wall thickness for Ks. Significant coefficients are shown in bold, and ns means non-significance. The models for Ks cannot be accepted as in both cases $P<0.05$.

Table S1 Overview of species, abbreviations, family, subfamily and genera of 28 conifer species in the Netherlands.

Table S2 Results of the structural equation models for the effects of pit and tracheid traits on cavitation resistance (|P50) (indicated in grey) and hydraulic conductivity (Ks) (indicated in green) shown in Fig. 6. Per relationship, standardized regression coefficients (beta), standard error (SE), Z-value, *P*-value and indirect effects are given. Bold fonts indicate significant regressions.

Table S3 Results of a multi-model comparison showing how hydraulic conductivity (Ks) depends on the tracheid traits of earlywood (in blue) and latewood (in green). Only the best (delta AIC<2) and averaged models were included. Bold fonts indicate significant coefficients. Adjusted R^2 (R^2_{adj}) and P values are shown.

Fig. S1 An example of a radial subsection (blue) from the cross-section for the measurements of *Tsuga heterophylla*. The tracheid diameter and wall thickness for earlywood (green) and latewood (black) were measured in this study.

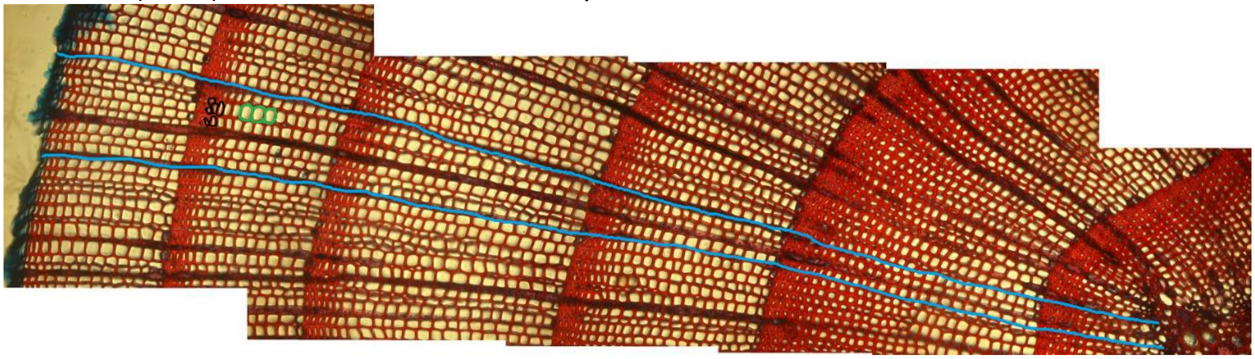

Fig. S2 Barplot of P50 (the xylem pressure when 50% of hydraulic conductance is lost) in and Ks (xylem specific hydraulic conductivity) across 28 conifer species (N=3-7 trees/species). Species with different letters are significantly different (Tukey HSD post hoc test, *P*<0.05). Error bars (± standard error of the mean value) are shown.

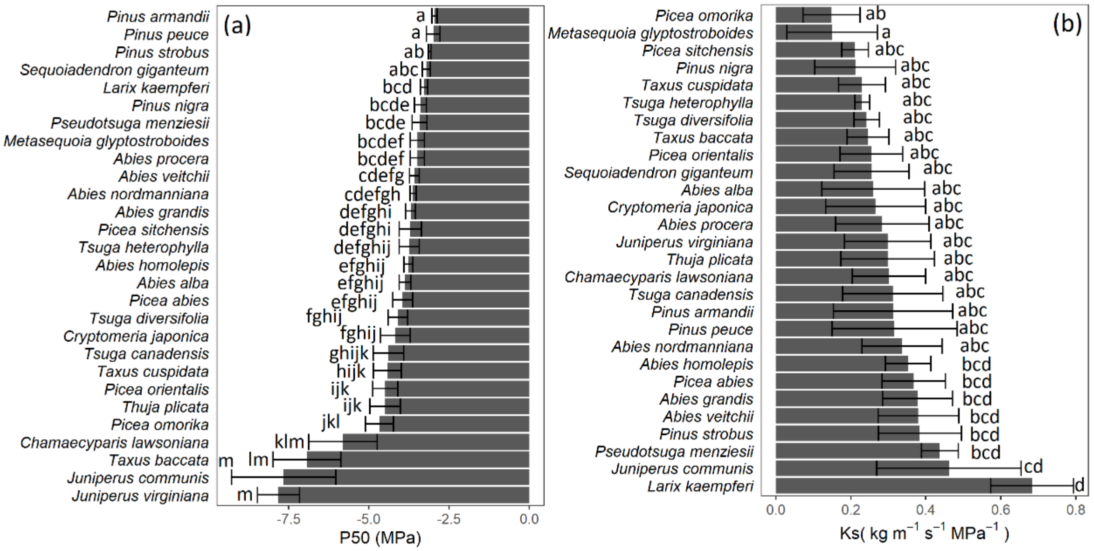

Fig. S3 Principal components analysis (PCA) of multivariate trait associations across 28 conifer species. The first two PCA axes and the loadings of 14 traits are shown. Different trait groups are indicated with different colored arrows for pit traits (red), earlywood traits (blue), latewood traits (green), average wood traits of early and latewood (purple), and hydraulic traits (black). Different families (Cupressaceae, Pinaceae, Taxaceae) are indicated by different symbols. For trait abbreviations (in Italics), see Table 1, for species abbreviations (in grey) see Table S1.

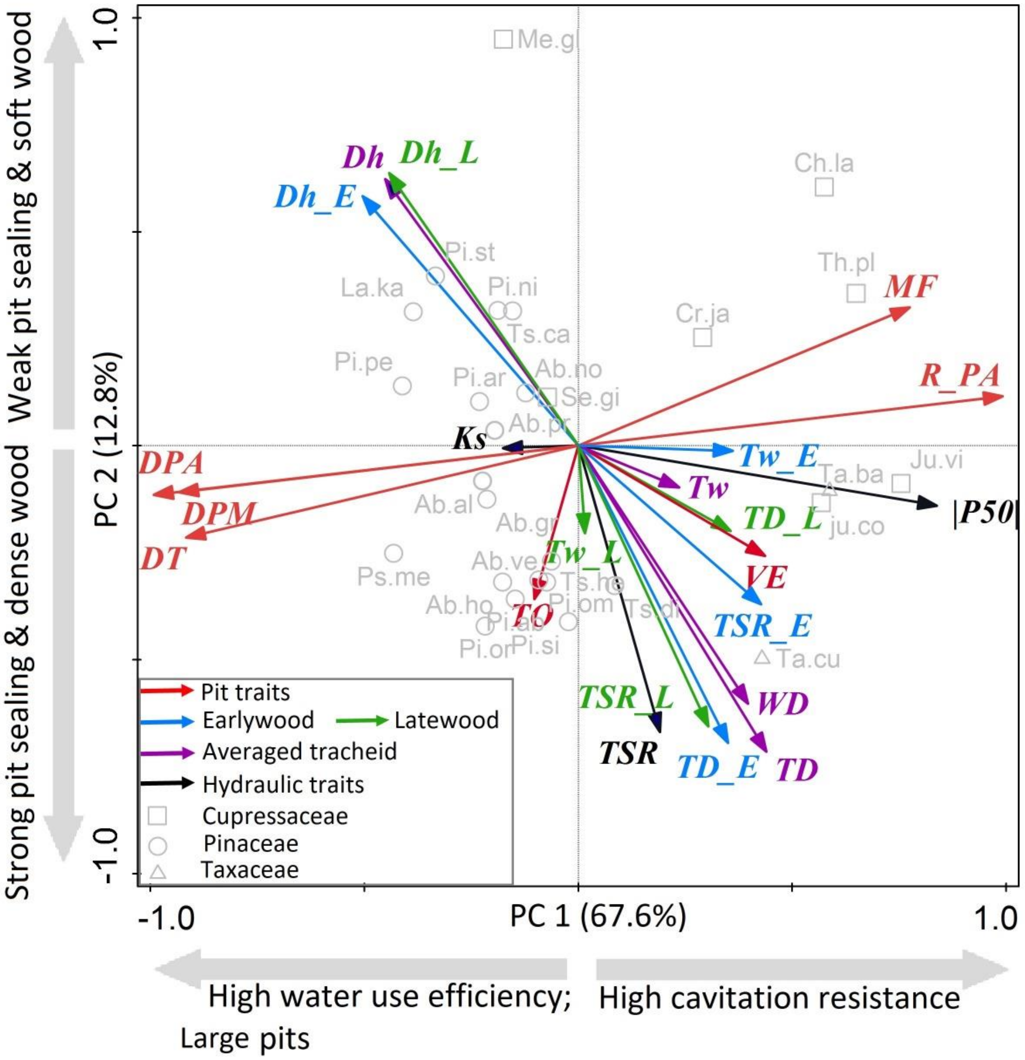

Fig. S4 Structural equation models for the effects of pit traits and tracheid traits of earlywood (a, c) and latewood (b, d) on the cavitation resistance (|P50|) and hydraulic conductivity (Ks) for 28 conifer species: (a) earlywood and |P50| (χ^2=23.38, df=17, *P*=0.053), (b) latewood and (|P50|) (χ^2=20.34, df=18, *P*=0.31), (c) earlywood and Ks (χ^2=19.18, df=7, *P*=0.01), (d) latewood and Ks (χ^2=15.76, df=7, *P*=0.03). The pit characteristics were all for earlywood, except pit aperture resistance could vary between early and latewood for P50; and tracheid diameter, density, and wall thickness for Ks. Significant coefficients are shown in bold, and ns means non-significance. The models for Ks cannot be accepted as in both cases *P*<0.05.

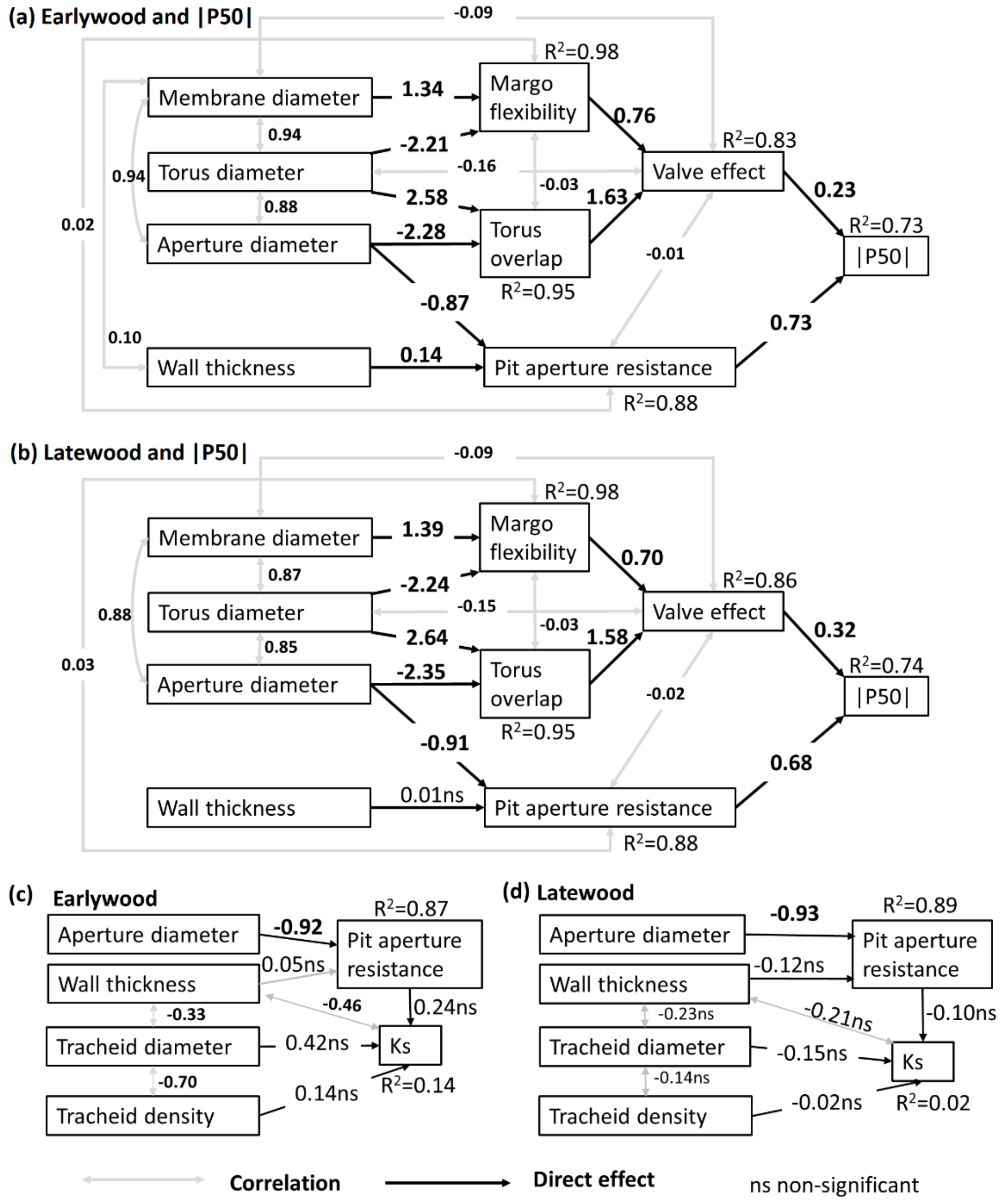

Table S1 Overview of species, abbreviations, family, subfamily and genera of 28 conifer species in the Netherlands.

Species	Abbreviation	Family	Subfamily	Genera
Abies alba	Ab.al	Pinaceae	Abietoideae	*Abies*
Abies grandis	Ab.gr	Pinaceae	Abietoideae	*Abies*
Abies homolepis	Ab.ho	Pinaceae	Abietoideae	*Abies*
Abies nordmanniana	Ab.no	Pinaceae	Abietoideae	*Abies*
Abies procera	Ab.pr	Pinaceae	Abietoideae	*Abies*
Abies veitchii	Ab.ve	Pinaceae	Abietoideae	*Abies*
Chamaecyparis lawsoniana	Ch.la	Cupressaceae	Cupressoideae	*Chamaecyparis*
Cryptomeria japonica	Cr.ja	Cupressaceae	Taxodioideae	*Cryptomeria*
Larix kaempferi	La.ka	Pinaceae	Laricoideae	*Larix*
Metasequoia glyptostroboides	Me.gl	Cupressaceae	Sequoioideae	*Metasequoia*
Juniperus communis	Ju.co	Cupressaceae	Cupressoideae	*Juniperus*
Juniperus virginiana	Ju.vi	Cupressaceae	Cupressoideae	*Juniperus*
Picea abies	Pi.ab	Pinaceae	Piceoideae	*Picea*
Picea orientalis	Pi.or	Pinaceae	Piceoideae	*Picea*
Picea omorika	Pi.om	Pinaceae	Piceoideae	*Picea*
Picea sitchensis	Pi.si	Pinaceae	Piceoideae	*Picea*
Pinus armandii	Pi.ar	Pinaceae	Pinoideae	*Pinus*
Pinus nigra	Pi.ni	Pinaceae	Pinoideae	*Pinus*
Pinus peuce	Pi.pe	Pinaceae	Pinoideae	*Pinus*
Pinus strobus	Pi.st	Pinaceae	Pinoideae	*Pinus*
Pseudotsuga menziesii	Ps.me	Pinaceae	Laricoideae	*Pseudotsuga*
Sequoiadendron giganteum	Se.gi	Cupressaceae	Sequoioideae	*Sequoiadendron*
Taxus baccata	Ta.ba	Taxaceae	-	*Taxus*
Taxus cuspidata	Ta.cu	Taxaceae	-	*Taxus*
Thuja plicata	Th.pl	Cupressaceae	Cupressoideae	*Thuja*
Tsuga canadensis	Ts.ca	Pinaceae	Abietoideae	*Tsuga*
Tsuga diversifolia	Ts.di	Pinaceae	Abietoideae	*Tsuga*
Tsuga heterophylla	Ts.he	Pinaceae	Abietoideae	*Tsuga*

Table S2 Results of the structural equation models for the effects of pit and tracheid traits on cavitation resistance (|P50) (indicated in grey) and hydraulic conductivity (Ks) (indicated in green) shown in Fig. 6. Per relationship, standardized regression coefficients (beta), standard error (SE), Z-value, *P*-value and indirect effects are given. Bold fonts indicate significant regressions.

Response variable	Predictor variable	beta	SE	Z	*P*
	**Direct effects**				
|P50|	Valve effect (VE)	**0.30**	0.10	3.01	0.003
	Pit aperture resistance (R_{PA})	**0.69**	0.11	5.99	<0.001
Valve effect	Torus overlap (TO)	**1.58**	0.07	22.29	<0.001
	Margo flexibility (MF)	**0.70**	0.09	7.72	<0.001
Torus overlap	Aperture diameter	**-2.34**	0.07	-33.20	<0.001
	Torus diameter	**2.64**	0.08	31.75	<0.001
Margo flexibility	Membrane diameter	**1.38**	0.05	28.95	<0.001
	Torus diameter	**-2.24**	0.05	-42.14	<0.001
Pit aperture resistance	Aperture diameter	**-0.93**	0.05	-17.45	<0.001
	Wall thickness	0.02	0.02	0.71	0.48
	**Indirect effects**				
Indirect effects from torus overlap via VE to |P50|					
|P50|	Torus overlap	**0.48**	0.16	2.98	0.003
Indirect effects from Margo flexibility via VE to |P50|					
|P50|	Margo flexibility	**0.21**	0.08	2.81	0.005
Indirect effects from aperture diameter via TO and R_{PA} to |P50|					
|P50|	Aperture diameter	**-1.75**	0.34	-5.12	<0.001
Indirect effects from wall thickness via R_{PA} to |P50|					
|P50|	Wall thickness	0.01	0.02	0.70	0.48
	**Direct effects**				
Ks	Hydraulic diameter	0.04	0.27	0.13	0.89
	Tracheid density	-0.13	0.25	-0.53	0.59
	Pit aperture resistance (R_{PA})	0.08	0.16	0.47	0.64
Pit aperture resistance	Aperture diameter	**-0.97**	0.06	-17.47	<0.001
	Wall thickness	-0.08	0.06	-1.44	0.15
	**Indirect effects**				
Indirect effects from aperture diameter via R_{PA} to Ks					
Ks	Aperture diameter	-0.07	0.16	-0.47	0.64
Indirect effects from wall thickness via R_{PA} to Ks					
Ks	Wall thickness	-0.01	0.01	-0.44	0.66

Table S3 Results of a multi-model comparison showing how hydraulic conductivity (Ks) depends on the tracheid traits of earlywood (in blue) and latewood (in green). Only the best (delta AIC<2) and averaged models were included. Bold fonts indicate significant coefficients. Adjusted R^2 (R^2_{adj}) and *P* values are shown.

model	Dh	TD	R_{PA}	Intercept	df	logLik	AICc	Weight	R^2_{adj}	*P*
Earlywood: Ks= D+TD+R_{PA}										
1				-1.05×10^{-16}	2	-39.22	82.90	0.42	0	1
2	0.20			1.80×10^{-17}	3	-38.64	84.30	0.21	<0.01	0.30
Avg	0.07									
Imp	0.34									
P	0.65									
Latewood: Ks= D+TD+R_{PA}										
1				-1.05×10^{-16}	2	-39.22	82.90	0.48		

Notes: Values indicate regression coefficients of the selected variables in the model. Per model, degrees of freedom (df), the log likelihood (logLik), corrected Akaike information criterion (AICc), and the AICc weight are given. Models are selected based on ΔAIC<2. The average model was calculated based on the selected models. The average coefficients (Avg), relative importance (Imp), and significances (*P*) are shown. Relative importance of the predictor variables is calculated as the sum of the Akaike weights over the best selected models. D, tracheid diameter; TD, tracheid density; R_{PA}, pit aperture resistance.
